# Structure-based activity prediction of CYP21A2 stability variants: A survey of available gene variations

**DOI:** 10.1038/srep39082

**Published:** 2016-12-14

**Authors:** Carlos D. Bruque, Marisol Delea, Cecilia S. Fernández, Juan V. Orza, Melisa Taboas, Noemí Buzzalino, Lucía D. Espeche, Andrea Solari, Verónica Luccerini, Liliana Alba, Alejandro D. Nadra, Liliana Dain

**Affiliations:** 1 Centro Nacional de Genética Médica, ANLIS, Buenos Aires, Argentina; 2 Instituto de Biología y Medicina Experimental, CONICET, Buenos Aires, Argentina; 3 Consultorio y Laboratorio de Genética, Rosario, Argentina; 4 Departamento de Química Biológica Facultad de Ciencias Exactas y Naturales, Universidad de Buenos Aires, IQUIBICEN-CONICET, Buenos Aires, Argentina

## Abstract

Congenital adrenal hyperplasia due to 21-hydroxylase deficiency accounts for 90–95% of CAH cases. In this work we performed an extensive survey of mutations and SNPs modifying the coding sequence of the *CYP21A2* gene. Using bioinformatic tools and two plausible CYP21A2 structures as templates, we initially classified all known mutants (n = 343) according to their putative functional impacts, which were either reported in the literature or inferred from structural models. We then performed a detailed analysis on the subset of mutations believed to exclusively impact protein stability. For those mutants, the predicted stability was calculated and correlated with the variant’s expected activity. A high concordance was obtained when comparing our predictions with available *in vitro* residual activities and/or the patient’s phenotype. The predicted stability and derived activity of all reported mutations and SNPs lacking functional assays (n = 108) were assessed. As expected, most of the SNPs (52/76) showed no biological implications. Moreover, this approach was applied to evaluate the putative synergy that could emerge when two mutations occurred *in cis.* In addition, we propose a putative pathogenic effect of five novel mutations, p.L107Q, p.L122R, p.R132H, p.P335L and p.H466fs, found in 21-hydroxylase deficient patients of our cohort.

Congenital adrenal hyperplasia (CAH) due to 21-hydroxylase deficiency (OMIM 201910) represents 90–95% of all CAH cases^1^,^2^,^3^. This autosomal recessive disorder, which is the most frequent inborn error of metabolism, has a broad spectrum of clinical forms, ranging from severe or classical, to mild late onset or non-classical (NC). The classical form includes the salt-wasting (SW) and simple virilizing (SV) of early onset. Girls with classical CAH are typically born with ambiguous genitalia. Patients with NC CAH exhibit clinical manifestations of hyperandrogenism.

The gene encoding 21-hydroxylase, *CYP21A2* is mapped to the short arm of chromosome 6 (6p21.3), within the human leukocyte antigen (HLA) complex, in the so-called RCCX module. Approximately two thirds of the chromosomes analyzed have a duplicated RCCX module that includes a genomic DNA segment composed of the pseudogenes *RP2, CYP21A1P, TNXA*, and a second active copy of the *C4* (long or short) gene^4^,^5^. *CYP21A1P* shares 98% sequence identity with *CYP21A2*. Due to the high degree of sequence identity between the gene and its pseudogene, most of the disease-causing mutations described in 21-hydroxylase deficiency are likely to be the consequence of non-homologous recombination or gene conversion events^6^,^7^. Although most of the patients carry *CYP21A1P*-derived mutations, an increasing number of naturally occurring mutations have been found in disease-causing alleles (see: http://www.hgmd.cf.ac.uk for details).

Mutations in the *CYP21A2* gene cause varying degrees of 21-hydroxylase activity loss. *In vitro* studies revealed that mutations leading to a complete inactivation of 21-hydroxylase are usually associated with the SW phenotype. Mutations that reduce enzyme activity close to 2% cause the SV phenotype, whereas those with a residual enzymatic activity in the range of 10% to 60% result in the mild NC CAH phenotype. In addition, a great number of patients are compound heterozygotes carrying different *CYP21A2* mutations on each allele, and their phenotypes depend on the milder gene defect^8^.

21-hydroxylase belongs to the cytochrome P450 protein family, a huge and diverse family found in bacteria, archaea and eukaryotes. In humans, there are 57 genes and more than 59 pseudogenes grouped in 18 families and 43 subfamilies, all with a high sequence identity^9^. 21-hydroxylase displays microsomal localization^10^ and, like other microsomal P450s, this enzyme accepts electrons provided by a NADPH-dependent P450 oxidoreductase (POR), reducing molecular oxygen and hydrolyzing substrates. This enzyme has 494–495 aminoacids with a molecular weight of 52 kDa^11^,^12^.

Over the last few years, much progress has been made towards predicting protein stabilities and correlating them to protein activities^13^,^14^,^15^,^16^,^17^. Homology modeling and fast energetic calculations have emerged as useful tools to evaluate, through structure-based methods, the impairment of protein stability. Human 21-hydroxylase models have been built based on the available low homology CYP protein families^13^,^16^.

With the aim of predicting the effect of newly uncharacterized mutations with improved accuracy, we have developed and evaluated a procedure based on the high identity bovine and human templates^18^,^19^. Using bioinformatic tools and either the human crystal structure or a model based on the bovine CYP21A2 counterpart, we initially classified all mutants in coding regions according to their putative role in protein dysfunction and/or location in the structure and focused our analysis on those affecting protein stability. Using this approach, we estimated *in silico* the residual activity of mutants that lack functional assays. Furthermore, we estimated the effect of double mutations/SNPs, located *in cis,* on P450CYP21 protein stability. In addition, we propose the putative pathogenic effect of five novel mutations, p.L107Q, p.L122R, p.R132H, p.P335L and p.H466fs, found in 21-hydroxylase deficient patients of our cohort.

## Results

### Survey of CYP21A2 reported variants

With the aim of predicting the effect of uncharacterized mutations, we initially performed an extensive survey of mutations and SNPs modifying the coding sequence of the gene (n = 343). Using either the human crystal structure (PDB ID:4Y8W) or a model based on the high identity crystal structure from the bovine protein (PDB ID:3QZ1), the variants were classified according to their proposed effect on protein dysfunction and/or location in the structure (Supplementary Information, Table S1).

### Correlation of protein stability and CYP21A2 activity

**Figure 1:**
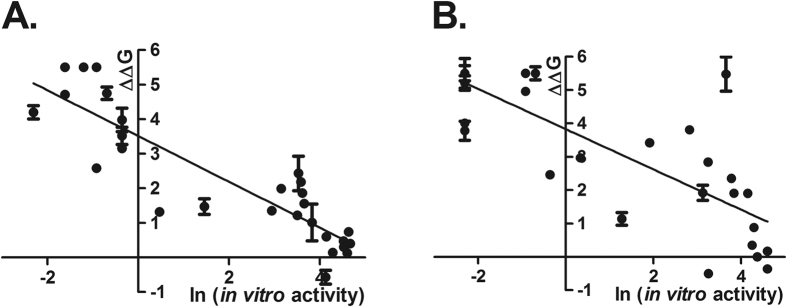
Correlation between experimental activities and predicted stabilities for Human and Bovine based templates. A total of 30 mutants in human P450CYP21A2 protein with *in vitro* functional studies published up to 2013 were analyzed using either our own generated three-dimensional structure of CYP21 based on the bovine template PDB ID: 3QZ1 (**A**) or the human crystal structure PDB ID: 4Y8W (**B**). Only residues evaluated to impair protein function by means of protein stability were included. The predicted free energy change (∆∆G) upon mutation was plotted against the natural logarithm (ln) of the residual enzymatic activity on 17-OHP (**A**) or Progesterone (**B**) as substrates. A value of 5, 5 kcal/mol was considered as the maximum one for the fitting as higher destabilization will not impair activity below 0%. Spearman’s correlation coefficients were −0.894 and −0.829 for the 3QZ1 model and 4Y8W, respectively. These values were statistically significant (p < 0.001, permutation test). The values of the regression line slopes were also statistically significant (p < 0.001) ΔΔG is expressed in kcal/mol.

We focused our analysis on mutations assumed to be involved in protein stability (148 variants), under the hypothesis that protein destabilization affects enzymatic activity. Of these, we initially selected variants with experimental enzymatic activity reported until 2013 (n = 30, see Table S1). Using the FoldX algorithm, the predicted free energy of each of the mutants relative to the wild type counterpart (∆∆G) was plotted against the natural logarithm (ln) of the *in vitro* activity as previously reported^16^. As shown in Fig. 1, a good correlation between FoldX’s predictions and experimental activity was obtained. Correlation was higher when using the bovine-based model (R^2^ = 0, 79) than for the human crystal structure (R^2^ = 0, 60). As a reference, we compared the *in vitro* activity for an overlapping set of mutants with the predicted stability reported by two previous models, obtaining a R^2^ = 0, 48 (n = 13) with the rabbit CYP2C5^13^ and a R^2^ = 0, 68 (n = 7) with the bovine CYP21A2^20^.

To validate our method, we estimated the *in silico* enzymatic activity of mutants with experimental activities reported from 2014 up to the present^21^,^22^,^23^,^24^, excluding residues known to impair protein function independently of protein stability. To accomplish this, the predicted residual activity after calculation of ∆∆G was compared with the activity reported in functional assays and with the patients’ phenotypes. As shown in Table 1, in 5 out of 10 mutations the predicted *in silico* residual activity was similar to the experimental results. Nevertheless, a close look at the remaining 5 mutations revealed that, contrary to the *in vitro* assay results, the *in* silico predicted p.P45L activity may correlate better with the patient’s phenotype (Table 1). For the p.K102R variant, the predicted activity using the bovine-based template might be related to a NC CAH allele. Nevertheless, the *in silico* activity predicted using the human crystal structure is consistent with the fact that p.K102R has long been considered a common polymorphism^25^. For the p.M150R variant, both computed *in silico* activities may predict severe alleles, whereas discordant results were reported *in vitro (*17%, NC; 4%, SV). Finally, for the p.M283V variant the values of the *in silico* activities were twice the residual enzymatic activities found *in vitro*. Nevertheless, both are activities found in mild alleles.

### *In silico* prediction of residual enzymatic activity

Using this procedure we predicted the *in silico* residual enzymatic activity of 32 CYP21A2 mutations^20^,^26^,^27^,^28^,^29^,^30^,^31^,^32^,^33^,^34^,^35^,^36^,^37^,^38^,^39^,^40^,^41^,^42^,^43^ that lack functional assays and are putatively involved in protein destabilization. The predicted activities were calculated from the linear fit based on the bovine template, which showed a better correlation with the experimental activities (see above). As shown in Table 2, in 10 mutations, the *in silico* activity was in accordance with the allele type expected from the observed phenotype. However, in 9 of 32 mutations such correlation could not be assessed due to the lack of information of the mutation in the homologous allele and/or the patient’s phenotype.

We extended our approach to the *CYP21A2* variants (n = 76) deposited as SNPs in public databases (Supplementary Information, Table S2). All the predicted enzymatic activities above 75% were considered to be non-pathogenic. Fifty-two SNPs disclosed no biological implications.

### Stability prediction of double mutants/SNPs located *in cis*

We aimed to identify CYP21A2 variants in which two mutations can combine to generate a severe effect and to identify variants presenting a synergistic effect, namely, improving or impairing the enzymatic activity to a greater extent than expected by the sum of each individual mutation. To this end, we analyzed the effect of two allelic variants (mutation-mutation, mutation-SNP or SNP-SNP) found *in cis* on protein stability.

Of all possible combinations for mutant-mutant or mutant-SNPs, we considered the following scenarios: 1) there is a trivial situation in which both mutations, as well as their addition, are above the cut-off value (1.6 kcal/mol), and consequently we expect a pathogenic effect (most cases reported, not shown); 2) another possibility is that neither of the mutations nor SNPs exceeds the cut off value but their sum indeed does, in which case we predict pathogenicity derived from impaired enzymatic activity (Supplementary Information, Tables S3A and S4A); 3) and finally, when only one of the mutations exceeds the cut off value but combined with another mutation or SNP their sum drops below the cut-off value, in which case we predict a non-pathogenic effect (Supplementary Information, Tables S3B and S4B).

Interestingly, FoldX allowed us to identify synergistic effects (either positive or negative) that may suggest a change in the classification of effect of the mutation. For example, a negative synergistic effect is one in which the sum of the effects of two mutations/SNPs exceeds the cut off, but their combined analysis by FoldX results in a lower value (Supplementary Information, Tables S3C,D and S4C,D), whereas a positive synergistic effect is one in which the sum of two mutations or SNPs does not exceed the cut-off value, but their combined analysis with FoldX does (Supplementary Information, Table S4E). Strikingly, there are cases in which neither of the mutations, nor their sum, exceeds the cut-off value, but FoldX nevertheless predicts a synergistic effect and thus, pathogenicity (Supplementary Information, Table S4F).

When SNP-SNP double mutants were analyzed, none of them presented values above the cut-off (not shown). The combination of p.K102R with p.S268T, each with a small contribution to destabilization (0.81 and 0.74 kcal/mol, respectively), did however result in a value close to that of the cut-off (1.55 kcal/mol).

### Structure-based predicted effect of novel mutants

We analyzed the putative structural and functional effects of 5 novel mutations, p.L107Q, p.L122R, p.R132H, p.P335L and p.H466fs, found in patients from our cohort (see Supplementary Information Table S5 for details on patients phenotypes and genotypes). None of these novel variants were found in the 1000 Genome Database. In addition, all but one, p.P335L, are located in CYP21 protein residues that are highly conserved throughout mammalian species (Supplementary Information, Figure S1).

**Figure 2:**
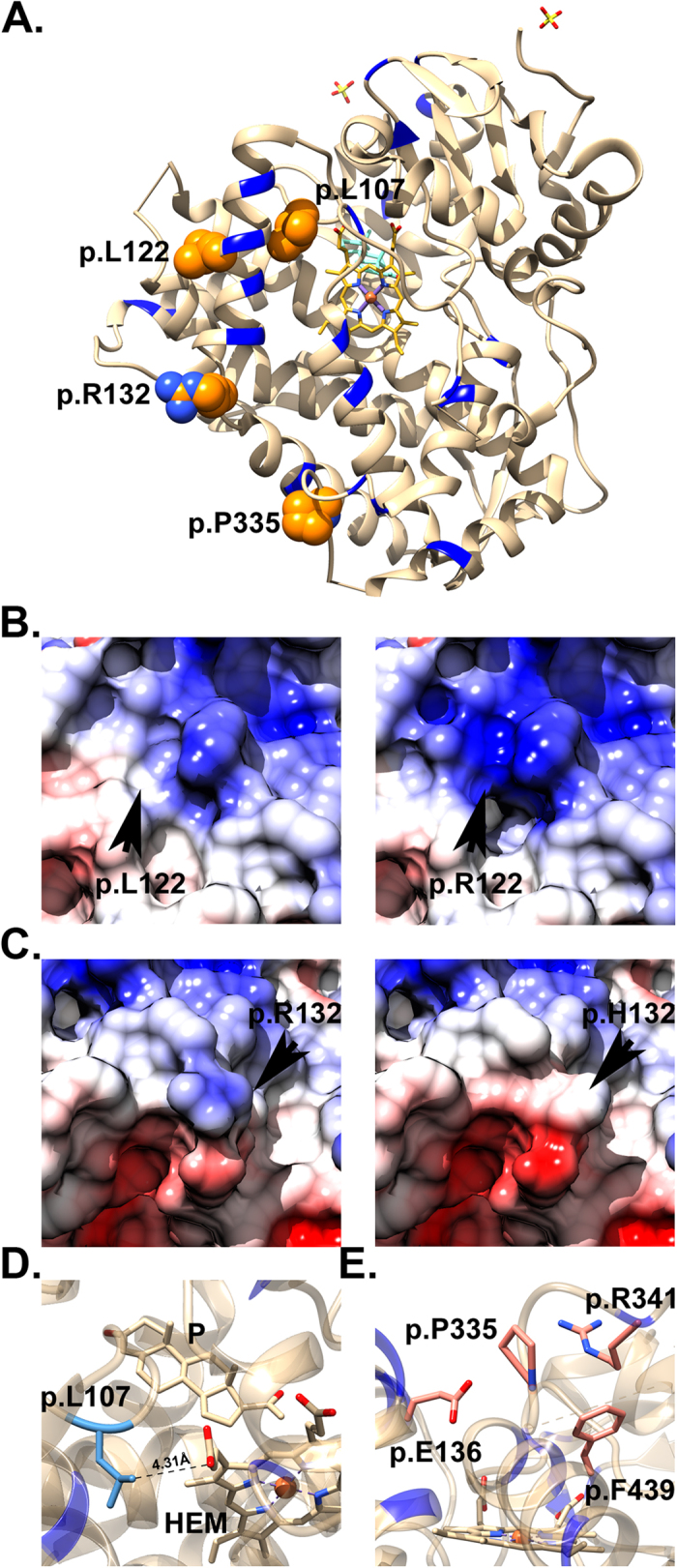
Structural analysis of novel point substitution in the human CYP21A2. (**A**) Cartoon representation of the residues involved in the novel point mutations found. Residues are labeled and highlighted by orange spheres. Heme cofactor is depicted in sticks. Residues involved in POR interaction are depicted in blue. (**B**) and (**C**) Differences in electrostatic surface upon mutations. Electrostatic surfaces of the wild type L122 and R122 as well as of the R122 and H132 mutants are represented. Residues are indicated by arrows. Acidic regions are depicted in red and basic ones in blue. (**D**) Cartoon representation of the residue L107 in the structure. Residue L107 (in light blue sticks) points towards the heme’s propionate moiety at a distance of 4.31 Å. HEM: heme group. P: Progesterone. (**E**) Cartoon representation of the residue P335 in the structure. Residues located nearby in the 3D structure are also shown.

Figure 2 shows the structural analysis of the novel point mutations on the human protein. As shown, the side chain of L107 is located very close (4.31Å) to the propionate moiety of the heme group (Fig. 2D). The introduction of glutamine’s positive charge might disrupt hydrophobic interactions that stabilize the heme group. The mutation in residue 122 replaces a hydrophobic leucine with a bulky and positively charged arginine residue, thus modifying the electrostatic potential surface of the protein. Conversely, the change of a positively charged arginine to a histidine residue in position 132 might cause a decrease in the density of positive charge on the protein’s surface (Fig. 2B,C). Both changes are positioned in regions where several amino acids have been suggested to interact with the POR^13^. Thus, we expect changes in electrostatic potential to significantly affect protein-protein interactions. Residue P335 (Fig. 2E) is located in a loop between helices K and L^19^. The change from a proline residue to a leucine does not introduce a charge modification in the region and neither heme/ligand nor POR interactions are involved, although a spatial displacement of the loop upon mutation cannot be ruled out. We classified this mutation as being putatively involved in protein stability. We found a ∆∆G of −2.16 ± 0.15 kcal/mol for the bovine-based template and consequently the *in silico* predicted activity is ≥100%. Similar results were obtained when modeling residues involved in novel point mutations using the bovine template (Supplementary Information, Figure S2).

Lastly, the p.H466fs mutation causes a frameshift in the carboxy-terminus of the protein, resulting in a completely different tract of 59 residues and introducing 27 additional amino acids to the protein. Consequently, this variant cannot be accurately modeled by the present approach; notwithstanding, a nonfunctional protein could be expected.

## Discussion

The adrenocortical 21-hydroxylase is one of the key enzymes in glucocorticoid and mineralocorticoid biosynthesis, and mutations in the *CYP21A2* gene cause the CAH as a result of 21-hydroxylase deficiency. Most of the reported mutations in the coding region result in aminoacid substitutions that may disturb essential functional and structural motifs of the protein (http://www.hgmd.cf.ac.uk). Activity impairment will also be evident when the mutation affects the correct folding and stability of the protein and thus its availability in the cell. Furthermore, an even more subtle case can be envisioned in which the protein’s activity is impaired by mutations that alter protein dynamics and thus, its behavior in the cell.

There are several examples of structure-based studies correlating specific aminoacidic change in CYP21A2 and other proteins with the severity of the encoded allele. CYP21A2 studies were initially based on low-identity templates^13^,^16^, and then repeated when a high-identity bovine protein^18^ was made available^20^. Recent publication of the human CYP21A2 crystal structure^19^ encouraged us to improve and expand our analysis by including this structure as template.

It is worth noting that most of our test data sets are obviously biased towards mutations with a deleterious effect as they come from clinical cases. For a subset of these mutations, functional assays have been performed, demonstrating their involvement in the pathogenicity of the disease. Nevertheless, for some of the reported variants, no information is available.

21-hydroxylase deficiency is a recessive disorder and most of the patients are compound heterozygotes with different mutations in each allele. Thus, a detailed description of the putative severity of a mutant protein must be carefully considered within the context of the mutation on the homologous chromosome and the patient’s phenotype.

Considering a protein length of 494–495 amino acids for the human CYP21 and only 20 different residues, the number of possible mutants is between 494^20^ and 495^20^. So far, only 210 of them have been seen in patients and, among these, approximately 33% are assumed to be involved in protein stability and could be evaluated by our method. In addition, 76 allelic variants presumably involved in protein destabilization were found in individuals from the general population, and their putative implications on protein activity are not known. We believe that activity prediction for variants could be useful to partially understand their pathophysiological implications. This is particularly relevant in the case of mild double mutants *in cis* in which the combined effect is unknown but may be predicted. In the future, this method could also be extended to several excluded positions depending on the development of algorithms capable of readily predicting heme and ligand interactions, and the availability of templates with different ligands (including interacting partners, such as POR). This sort of extension has been implemented to successfully assess protein-DNA interactions^15^,^44^.

It is expected that both the sequence identity and structural resolution improve energetic predictions. Surprisingly, in our analysis, stability calculations based on the bovine crystal structure resulted in a better correlation to experimental measurements than those based on the human counterpart. We hypothesize that these unexpected results could be related to the experimental conditions in which each structure was obtained. X-Ray diffraction crystallography provides a picture of the lowest energy conformation for a given protein, usually biasing our interpretation to a unique and rigid entity. Furthermore, proteins that interact with several ligands often adopt different conformations depending on the binding partner. In such cases, it is useful to have several structures with the different ligands or NMR data to further understand the conformational states of the system. In our study we have worked with only two structures: the bovine protein bound to 17-hydroxyprogesterone (17-OHP) and the human CYP21A2 bound to progesterone, each of which may represent a biased conformation that may in turn affect the structure-based energetic calculations. Thus, considering that proteins are dynamic entities that fluctuate and interact with several ligands in the cell, the overall behavior may be better grasped by a structure that could represent protein fluctuations around the energetic minimum rather than a structure representing the minimum itself as observed in the static crystal.

Remarkably, when we validated our method taking into account the most recent mutations with functional assays reported, half of them were in agreement with *in vitro* residual activities. Only 1 out of the 10 variants, p.R149C, was found to be a completely discordant result, and contrary to the functional assays, some of the *in silico* results may better represent the patient’s phenotype.

We then proceeded to predict stability and associated activity for all reported mutations or SNPs not expected to be involved in other processes except for protein stability. As expected, most of the SNPs described in population-based studies were found to have no biological implications. Moreover, when a correlation of the *in silico* results and the expected activity from the observed phenotype could be assessed, we found consistency in 10 of the variants analyzed. These results reinforce our approach as a useful tool for predicting residual activities of uncharacterized allelic variants.

A number of 21-hydroxylase deficient alleles have been reported with two mutations occurring *in cis*. Thus, we extended our method to analyze CYP21A2 variants that can combine to generate a severe and/or synergistic effect, including those reported to have no biological effect on protein activity (SNPs). Though there are very few experimental results of the final residual activity of *in-cis* double mutants to compare with, we predicted several combinations to have a severe and/or synergistic effect of a rather small magnitude. Strikingly, the combination of p.K102R with p.S268T, two variants classified individually as non-pathogenic in experimental assays^24^,^45^ reaches a destabilization value close to the cut-off. This is particularly important, since both variants are described in a great number of individuals from the general population^46^. Though the absence of mutations in patients diagnosed with 21-hydroxylase deficiency has been described previously, the putative pathogenic effect of two SNPs *in cis* has yet to be considered. Indeed, several authors have suggested that p.S268T could result in a decreased enzymatic activity when presented *in cis* with another polymorphic variant^47^,^48^.

The structure-based approach enables us the prediction of the effect of mutations that modify protein stability. For mutations impairing activity by other means, it is necessary to perform *in vitro* activities or develop computational tools that can explicitly model interactions with ligands (e.g. substrates, the heme group or other proteins). From the set of newly described mutants, only p.P335L was expected to affect stability. Nevertheless, we found that the predicted activity of this variant should be close to that of the wild-type protein since no destabilization change was found. Sequence comparisons demonstrated variability in the 335 residue throughout CYP21 proteins of different mammalian species. Indeed, a leucine residue is located at this position in the mouse and the rat proteins. In addition, *in vitr*o studies have suggested that the presence of two mild mutations *in cis* is generally associated with a severe impairment of enzymatic activity^23^,^49^,^50^,^51^. However, the patient with the p.P335L variant presented a NC phenotype, carrying a large gene conversion/deletion of the *CYP21A2* gene on the homologous allele (null allele) and the mild p.V281L mutation *in cis.* Taken together, these observations prompted us to suggest that this allelic variant may not influence the residual activity of the protein, in agreement with the *in silico* predictions. However, caution should be taken considering that a significant stabilization was found for this variant and a large stabilization could also affect protein degradation or its dynamics, and thus protein function.

In the post-genomic and personalized medicine era a large amount of genetic information is expected to accumulate. The development of an efficient tool to analyze this information is of utmost importance. In particular, connecting sequence information to phenotypic effects is an ongoing effort that could assist physicians in the near future. So far, most of the information on mutants is biased towards pathological effects but sooner or later an immense amount of uncharacterized variants will be described with the massive sequencing approaches currently underway. Until statistics or other methods can be used with enough confidence, we propose that *in silico* activity prediction using structure-based analysis could be a valuable tool, being particularly relevant in the case of double variants occurring *in cis*.

## Materials and Methods

### Ethical Approval

All the procedures performed in this study were in accordance with the ethical standards of the institutional and/or national research committee and with the 1964 Helsinki declaration and its later amendments or comparable ethical standards. Written informed consent was obtained from all patients and parents involved in this work. The study was approved by the ethics committee of the Administración Nacional de Laboratorios e Institutos de Salud (ANLIS), Buenos Aires, Argentina.

### *CYP21A2* genotyping

Details on methods in mutation genotyping and sequence alignments are presented in Supplementary Information.

### Survey of allelic variants and mutations in human CYP21A2

Mutations and allelic variants in the coding region of the CYP21A2 were extracted from the Human Cytochrome P450 Allele Nomenclature database (www.cypalleles.ki.se), and from the bibliography. In addition, database of single nucleotide polymorphisms (SNPs) and multiple small-scale variations that include insertions/deletions, microsatellites and non-polymorphic variants (http://www.ncbi.nlm.nih.gov/projects/SNP/)^52^, as well as the 1000 Genome Database^53^ were also consulted. When available, the *in vitro* activity for progesterone (P) and/or 17-hydroxyprogesterone (17-OHP) were included. When more than one activity was reported for the same mutation, we considered references that provided measurements of residual activity exclusively in *ex vivo* systems (in COS1 or COS7 cells). In addition, when possible, the most accurate, newest and better related to patient’s phenotype was preferred. The full list of mutations/SNPs considered is listed in Supplementary Information Table S1.

### Model building and assessment

In addition to the human crystal (PDB ID: 4Y8W), a model of the human CYP21A2 protein based on the structure of the bovine CYP21A2 (PDB ID: 3QZ1) was generated using MODELLER version 9.11^54^ including heme cofactor explicitly. Amino acid sequence was taken from UniProt (NP_000491.4) while alignments were performed with MEGA 4 software^55^. A significant improvement of the model was obtained by manually displacing L129 in the automatic alignment and iterative loop refinement. The use of multiple templates of other proteins of the CYP family did not improve the model (not shown). Model’s quality was assessed by DOPE^56^, QMEAN Z-scores^57^ and Ramachandran plots^58^. Protein model is available at www.modelarchive.org/project/index/doi/ma-anifj.

### *In silico* mutagenesis

Mutations were generated and analyzed using FoldX 3.0 Beta 5.1 (foldx.crg.es)^59^. Repair PDB FoldX command was used to optimize the total energy of the protein to FoldX’s force field before mutations were done. Mutagenesis was carried out using the BuildModel FoldX command, and each mutation was calculated five times.

### Variant classification

Variants were classified according to the following categories: nonsense, indel, deletion or duplication, heme or ligand interaction, POR interaction, protein degradation, Meander and the ERR-Triad, or stability (Table S1). For those in the latter group, we proceeded to calculate protein stabilities and correlated them with reported *in vitro* activities (see below). Some residues were not classified into the former categories, but were excluded instead due to a poor structure resolution of the corresponding templates.

Residues were considered to be involved in heme or ligand binding when the amino acid is within 5 Angstroms and pointing towards heme/ligand. Residues were considered to interact with POR according to Robins *et al*.^13^, or when charged residues (Arginine and Glutamic acid) were found in a close proximity and exposed to the same surface than those reported by Robins *et al*.^13^, namely residues R124, E140, E320, R341, R356, R366, R369, R431 and R444.

### Stability calculations

Protein stabilities were calculated using FoldX’s Stability command, and ∆∆G values were estimated as the difference between the energy of the wild type protein and the average of five replicas for each point mutation. A threshold of 1, 6 kcal/mol was considered, as it corresponds to twice the standard deviation calculated with FoldX. We considered values above this threshold to significantly destabilize a protein. Mutations located in functional residues (e.g. those involved in disulfide bridge or in heme, substrate or ligand binding) were excluded from the stability analysis as their effects on protein activity are influenced by other variables besides protein stability. To favor wild type conformation, all residues involved in ligand or heme interactions where fixed when optimizing the structures to FoldX force field.

### Statistical analyses

The predicted free energy of each of the mutants relative to the wild type counterpart (∆∆G) was plotted against the natural logarithm (ln) of the *in vitro* activity. Mutants with experimental activities around 0% of the wild type presented destabilization values around 5, 5 kcal/mol. Thus, this value was considered as the maximum one for the fitting, even when FoldX predicts larger values (mutants L142P and L166P). The predicted activities of those mutants lacking functional assays were derived from the fitting of the abovementioned correlation.

Statistical analyses were performed using the Infostat V.11 (http://www.infostat.com.ar/?lang=en) and GraphPad Prism V. 5.01 Softwares (http://www.graphpad.com/scientific-software/prism/). Spearman’s correlation coefficient was used to determine a monotonous relation between ∆∆G and the ln of the activity. Permutation test was applied to evaluate its statistical significance. The parameters of the linear regression were calculated by the least squares method and the statistical significance of the regression line’s slope by a permutation test. A p < 0.05 was considered significant.

### Double mutant/SNP effect

Only those variants with a reported *in vitro* activity and classified as putatively involved in protein stability were analyzed. Double mutants/SNPs *in cis* were generated as described above. Synergistic effect in those cases was assessed by comparing the estimated ∆∆G of the double mutant to the sum of both single mutants. When there is no synergy, the difference between them tends to zero.

## Additional Information

**How to cite this article**: Bruque, C. D. *et al*. Structure-based activity prediction of CYP21A2 stability variants: A survey of available gene variations. *Sci. Rep.* **6**, 39082; doi: 10.1038/srep39082 (2016).

**Publisher's note:** Springer Nature remains neutral with regard to jurisdictional claims in published maps and institutional affiliations.

## Supplementary Material

Supplementary Information

## Figures and Tables

**Table 1:** Comparison of *in vitro* and *in silico* predicted activities of aminoacidic variants in CYP21A2 presumed to be involved in protein stability published since 2014.

Mutation	*In vitro* activity ± SD		*In silico* activity		Patient’s second mutation (REA%)	Patient’s phenotype	Reference
	17- OHP	P	Bovine model	Human crystal structure			
p.P45L	105 ± 10	ND	0.74	1.46	ND	SV	21
p.K102R	119.7 ± 25	ND	63.16	≥100	ND	NA	21
p.L122P	1.4 ± 2.1	−1.9 ± 5.2	0.11	0.25	Deletion (0)	SW	22
p.R149C	35.8 ± 14.6	47.3 ± 12.9	≥100	≥100	p.V281L (60)	NC	23
p.M150R	17.7 ± 1.9	4.6 ± 1.9	0.9	4.4	N	PB	22
p.A159T	126.6 ± 29.9	ND	≥100	≥100	N	AD	21
p.V211M	99.5 ± 32.4	ND	≥100	≥100	ND	SV/NC	21
p.A265S	90 ± 9	104 ± 15	≥100	≥100	N	NA	24
p.M283V	16.2 ± 9.3	19 ± 7	41.8	38.14	Deletion (0)	NC	23
p.M473I	85 ± 7	66 ± 12	≥100	64.12	p.V281L (60)	NC	24

*In silico* enzymatic activities were calculated from the fitting of the bovine based model and from the human crystal (Fig. 1) using the estimated **∆∆**Gs of each of the mutants (Table S1). *In vitro* and *in silico* enzymatic activities are expressed in percentage relative to the wild type protein considered as 100%. *In vitro* enzymatic activities, patients’ second mutation and phenotypes are those reported in the bibliography. 17-OHP: 17-hydroxyprogesterone. P: Progesterone. REA: Residual enzymatic activity. ND: Not determined. N: Normal; NC. Nonclassical; SV: Simple virilizing; SW: Salt wasting; NA: Not Affected; PB: Premature Pubarche; AD: Addison Disease.

**Table 2:** *In silico* predicted residual activity of CYP21A2 stability mutants lacking functional assays.

Variant	Patient’s second mutation (% REA)	Patient’s phenotype	Expected activity of the variant	∆∆G	*In silico* activity (%)	Reference
p.H38L	NA	NA	?	−1.64	100	26
p.Y47C	NA	NA	?	−1.64	100	27
p.Y59N	NA	CL	0–5	2.54	4.4	28
p.V69L	p.[Q318;.R356] (0) pH62L in cis*	SV	5–60	1.47	22.5	29
p.S113Y	p.V281L (60)	NC	0–60	0.79	63.3	30
p.S113F	NA	NA	?	1.05	42.6	20
p.Q144P	p.P482fs (ND)	SW	0	0.61	83.2	29
p.F164S	p.I172N (2)	SV	0–5	1.37	26.2	29
p.S165P	p.I172N (2)	NC	10–60	4.94	0.1	31
p.T168N	Chimeric gene (0)	NC	10–60	3.41	1.2	32
p.V211L	NA	N?	?	−0.91	100	33
p.E238K	NA	4 SW, 1 NC	0^**#**^	−0.33	100	34
p.V249A	NA	N	100	0.45	100	35
p.L261P	c.290-13A/C>G^**^ (0–2)	SW	0	8.95	0.01	36
p.M283L	p.V281L (60)	NC	0–60	−0.3	100	37
p.S301Y	c.290-13A/C>G^**^ (0–2)	NC	10–60	12.31	0.00	38
p.V304E	c.290-13A/C>G^**^ (0–2)	SW	0	2.44	5.1	29
p.V305D	NA	NA	?	0.99	46.6	20
p.F306V	NA	NA	?	1.87	12.2	20
p.L307V	Deletion (0)	NC	10–60	2.8	2.9	30
p.R316L	p.V281L (60)	NC	0–60	0.03	100	30
p.L317M	c.290-13A/C>G^**^ (0–2)	NC	10–60	−0.8	100	39
p.L317V	N	NC^**§**^	?	2.42	5.3	40
p.L321P	NA	NA	?	9.84	0.0	20
p.G381S	NA	NA	?	3.51	1.0	20
p.N387K	p. V281L (60)	NC	0–60	7.55	0.0	41
p.F404S	p.F404S (ND)	SW	0	5.34	0.06	42
p.F404L	p.V281L (60)	NC	0–60	2.51	4.6	30
p.T450P	p.T450P (ND)	SW	0	9.35	0.0	42
p.P459H	p.[ClEx6; Q318X; A391T] (0)	SV	2–5	4.44	0.2	43
p.P459S	Chimeric Gene	SV	2–5	2.68	3.6	32
p.P459L	c.290-13A/C>G^**^ (0–2)	SV	0–5	1.29	29.5	29

*In silico* predicted activities of mutants lacking functional assays were compared with the expected ones. Expected activity was established considering the residual activity of the mutation on the homologous allele and/or the patient’s phenotype when available. *In silico* enzymatic activities were calculated from the fitting based on the bovine template using the estimated **∆∆**G of each of the variants (Table S1). Activities are expressed relative of the wild type protein (100%). *According to functional assays, p.H62L mutation was classified as a mild mutation. Nevertheless, several alleles were described having another mild mutation *in cis* with decreased enzymatic activities most likely related to the SV form of the disease^50^,^60^. ^**^c.290-13A/C>G mutation creates a new acceptor splice site. Patients bearing this mutation have been described presenting either a SW or a SV phenotype^8^. ^#^The classification of the expected activity for this variant was based on the fact that 4/5 patients presented a SW phenotype. ^**§**^The patient described by Byounga *et al*.^40^, disclosed a 17-OHP post ACTH value of 6, 67 ng/mL. According to the current inclusion criteria, patients would be classified as presenting a NC form of the disease when the post ACTH test is at least 10 ng/mL^61^. REA: Residual enzymatic activity. NA: Not available, ND: Not determined; N. Normal; NC. Nonclassical; SV: Simple virilizing; SW: Salt wasting; CL: Classical; N: Normal; ClEx6: Cluster Exon 6 mutations. ?: Insufficient data to estimate the expected enzymatic activity.

## References

[b1] New M. I., White P. C., Pang S. Dupont B. & Speiser P. W. The Adrenal Hyperplasias in The Metabolic Basis of Inherited Disease (eds Scriver C. R., Beaudet A. L., Sly S. & Valle D.) 1881–1917 (McGraw-Hill, 1989).

[b2] Miller W. L. Clinical review 54: Genetics, diagnosis, and management of 21-hydroxylase deficiency. J. Clin. Endocrinol. Metab. 78, 241–246 (1994). 8106606 10.1210/jcem.78.2.8106606

[b3] Pang S. & Shook M. K. Current status of neonatal screening for congenital adrenal hyperplasia. Curr. Opin. Pediatr. 9, 419–423 (1997). 9300201 10.1097/00008480-199708000-00018

[b4] Koppens P. F. . Family studies of the steroid 21-hydroxylase and complement C4 genes define 11 haplotypes in classical congenital adrenal hyperplasia in The Netherlands. Eur. J. Pediatr. 151, 885–892 (1992). 1473541 10.1007/BF01954123

[b5] Blanchong C. A. . Deficiencies of human complement component C4A and C4B and heterozygosity in length variants of RP-C4-CYP21-TNX (RCCX) modules in caucasians. The load of RCCX genetic diversity on major histocompatibility complex-associated disease. J. Exp. Med. 191, 2183–2196 (2000). 10859342 10.1084/jem.191.12.2183 PMC2193198

[b6] Donohoue P. A. . Gene conversion in salt-losing congenital adrenal hyperplasia with absent complement C4B protein. J. Clin. Endocrinol. Metab. 62, 995–1002 (1986). 3007562 10.1210/jcem-62-5-995

[b7] Higashi Y., Tanae A., Inoue H. & Fujii-Kuriyama Y. Evidence for frequent gene conversion in the steroid 21-hydroxylase P-450(C21) gene: implications for steroid 21-hydroxylase deficiency. Am. J. Hum. Genet. 42, 17–25 (1988). 2827462 PMC1715324

[b8] White P. C. & Speiser P. W. Congenital adrenal hyperplasia due to 21-hydroxylase deficiency. Endocr. Rev. 21, 245–291 (2000). 10857554 10.1210/edrv.21.3.0398

[b9] Nelson D. R. . Comparison of cytochrome P450 (CYP) genes from the mouse and human genomes, including nomenclature recommendations for genes, pseudogenes and alternative-splice variants. Pharmacogenetics 14, 1–18 (2004). 15128046 10.1097/00008571-200401000-00001

[b10] Kominami S., Ochi H., Kobayashi Y. & Takemori S. Studies on the steroid hydroxylation system in adrenal cortex microsomes. Purification and characterization of cytochrome P-450 specific for steroid C-21 hydroxylation. J. Biol. Chem. 255, 3386–3394 (1980). 6767716

[b11] Higashi Y., Yoshioka H., Yamane M., Gotoh O. & Fujii-Kuriyama Y. Complete nucleotide sequence of two steroid 21-hydroxylase genes tandemly arranged in human chromosome: a pseudogene and a genuine gene. Proc. Natl. Acad. Sci. USA 83, 2841–2845 (1986). 3486422 10.1073/pnas.83.9.2841 PMC323402

[b12] White P. C., New M. I. & Dupont B. Structure of human steroid 21-hydroxylase genes. Proc. Natl. Acad. Sci. USA 83, 5111–5115 (1986). 3487786 10.1073/pnas.83.14.5111 PMC323900

[b13] Robins T., Carlsson J., Sunnerhagen M., Wedell A. & Persson B. Molecular model of human CYP21 based on mammalian CYP2C5: structural features correlate with clinical severity of mutations causing congenital adrenal hyperplasia. Mol. Endocrinol. 20, 2946–2964 (2006). 16788163 10.1210/me.2006-0172

[b14] Pey A. L., Stricher F., Serrano L. & Martinez A. Predicted effects of missense mutations on native-state stability account for phenotypic outcome in phenylketonuria, a paradigm of misfolding diseases. Am. J. Hum. Genet. 81, 1006–1024 (2007). 17924342 10.1086/521879 PMC2265664

[b15] Alibés A. . Using protein design algorithms to understand the molecular basis of disease caused by protein-DNA interactions: the Pax6 example. Nucleic Acids Res. 38, 7422–7431 (2010). 20685816 10.1093/nar/gkq683 PMC2995082

[b16] Minutolo C. . Structure-based analysis of five novel disease-causing mutations in 21-hydroxylase-deficient patients. PLoS One 6, e15899 (2011). 21264314 10.1371/journal.pone.0015899 PMC3019215

[b17] Worth C. L., Preissner R. & Blundell T. L. SDM–a server for predicting effects of mutations on protein stability and malfunction. Nucleic Acids Res. 39, W215–222 (2011). 21593128 10.1093/nar/gkr363 PMC3125769

[b18] Zhao B. . Three-dimensional structure of steroid 21-hydroxylase (cytochrome P450 21A2) with two substrates reveals locations of disease-associated variants. J. Biol. Chem. 287, 10613–10622 (2012). 22262854 10.1074/jbc.M111.323501 PMC3323056

[b19] Pallan P. S. . Human Cytochrome P450 21A2, the Major Steroid 21-Hydroxylase: Structure of the enzyme progesterone substrate complex and rate-limiting C-H bond cleavage. J. Biol. Chem. 290, 13128–13143 (2015). 25855791 10.1074/jbc.M115.646307 PMC4505568

[b20] Haider S. . Structure-phenotype correlations of human CYP21A2 mutations in congenital adrenal hyperplasia. Proc. Natl. Acad. Sci. USA 110, 2605–2610 (2013). 23359706 10.1073/pnas.1221133110 PMC3574933

[b21] Brønstad I. . Functional studies of novel CYP21A2 mutations detected in Norwegian patients with congenital adrenal hyperplasia. Endocr. Connect. 3, 67–74 (2014). 24671123 10.1530/EC-14-0032 PMC3987286

[b22] Massimi A. . Functional and Structural Analysis of Four Novel Mutations of CYP21A2 Gene in Italian Patients with 21-Hydroxylase Deficiency. Horm. Metab. Res. 46, 515–520 (2014). 24799024 10.1055/s-0034-1371864

[b23] Taboas M. . Functional studies of p.R132C, p.R149C, p.M283V, p.E431K, and a novel c.652-2A>G mutations of the CYP21A2 gene. PLoS One 9, e92181 (2014). 24667412 10.1371/journal.pone.0092181 PMC3965420

[b24] Barbaro M. . *In vitro* functional studies of rare CYP21A2 mutations and establishment of an activity gradient for nonclassic mutations improve phenotype predictions in congenital adrenal hyperplasia. Clin. Endocrinol. (Oxf). 82, 37–44 (2014). 24953648 10.1111/cen.12526

[b25] Rodrigues N. R. . Molecular characterization of the HLA-linked steroid 21-hydroxylase B gene from an individual with congenital adrenal hyperplasia. EMBO J. 6, 1653–1661 (1987). 3038528 10.1002/j.1460-2075.1987.tb02414.x PMC553538

[b26] Tardy V. Gene symbol: CYP21A2. Disease: steroid 21-hydroxylase deficiency. Hum. Genet. 119, 363 (2006). 17230656

[b27] Tardy V. T. V. & Morel Y. Gene symbol: CYP21A2. Hum. Genet. 121, 293 (2007). 17598237

[b28] Tardy V. T. V. Gene symbol: CYP21A2. Hum. Genet. 121, 292–293 (2007). 17598236

[b29] Wang R. . 21-Hydroxylase deficiency-induced congenital adrenal hyperplasia in 230 Chinese patients: Genotype–phenotype correlation and identification of nine novel mutations. Steroids 108, 47–55 (2016). 26804566 10.1016/j.steroids.2016.01.007

[b30] New M. I. . Genotype-phenotype correlation in 1,507 families with congenital adrenal hyperplasia owing to 21-hydroxylase deficiency. Proc. Natl. Acad. Sci. USA 110, 2611–2616 (2013). 23359698 10.1073/pnas.1300057110 PMC3574953

[b31] Milacic I. . Molecular genetic study of congenital adrenal hyperplasia in Serbia: novel p.Leu129Pro and p.Ser165Pro CYP21A2 gene mutations. J. Endocrinol. Invest. 38, 1199–1210 (2015). 26233337 10.1007/s40618-015-0366-8

[b32] Vrzalová Z. . Identification of CYP21A2 mutant alleles in Czech patients with 21-hydroxylase deficiency. Int. J. Mol. Med. 26, 595–603 (2010). 20818501 10.3892/ijmm_00000504

[b33] Speiser P. W., New M. I. & White P. C. Molecular Genetic Analysis of Nonclassic Steroid 21-Hydroxylase Deficiency Associated with HLA-B14, DR1. N. Engl. J. Med. 319, 19–23 (1988). 3260007 10.1056/NEJM198807073190104

[b34] Kirac D. . The Frequency and the Effects of 21-Hydroxylase Gene Defects in Congenital Adrenal Hyperplasia Patients. Ann. Hum. Genet. 78, 399–409 (2014). 25227725 10.1111/ahg.12083

[b35] Concolino P., Mello E., Zuppi C. & Capoluongo E. Molecular diagnosis of congenital adrenal hyperplasia due to 21-hydroxylase deficiency: an update of new CYP21A2 mutations. Clin. Chem. Lab. Med. 48, 1057–1062 (2010). 20482300 10.1515/CCLM.2010.239

[b36] Loke K. Y., Lee Y. S., Lee W. W. & Poh L. K. Molecular analysis of CYP-21 mutations for congenital adrenal hyperplasia in Singapore. Horm. Res. 55, 179–84 (2001). 11598371 10.1159/000049992

[b37] Ezquieta B. . Non-classical 21-hydroxylase deficiency in children: association of adrenocorticotropic hormone-stimulated 17-hydroxyprogesterone with the risk of compound heterozygosity with severe mutations. Acta Paediatr. 91, 892–898 (2002). 12222711 10.1080/080352502760148595

[b38] Stikkelbroeck N. M. M. L. . CYP21 gene mutation analysis in 198 patients with 21-hydroxylase deficiency in The Netherlands: six novel mutations and a specific cluster of four mutations. J. Clin. Endocrinol. Metab. 88, 3852–3859 (2003). 12915679 10.1210/jc.2002-021681

[b39] Deneux C. . Phenotype-genotype correlation in 56 women with nonclassical congenital adrenal hyperplasia due to 21-hydroxylase deficiency. J. Clin. Endocrinol. Metab. 86, 207–213 (2001). 11232002 10.1210/jcem.86.1.7131

[b40] Bojunga J. . Structural and functional analysis of a novel mutation of CYP21B in a heterozygote carrier of 21-hydroxylase deficiency. Hum. Genet. 117, 558–564 (2005). 16028060 10.1007/s00439-005-1339-3

[b41] Wasniewska M. . Novel mutation of CYP21A2 gene (N387K) affecting a non-conserved amino acid residue in exon 9. J Endocrinol Invest 32, 633 (2009). 19474522 10.1007/BF03346522

[b42] Baradaran-Heravi A. . Three novel CYP21A2 mutations and their protein modelling in patients with classical 21-hydroxylase deficiency from northeastern Iran. Clin. Endocrinol. (Oxf). 67, 335–41 (2007). 17573904 10.1111/j.1365-2265.2007.02886.x

[b43] Jiang L. . Identification and functional characterization of a novel mutation P459H and a rare mutation R483W in the CYP21A2 gene in two Chinese patients with simple virilizing form of congenital adrenal hyperplasia. J. Endocrinol. Invest. 35, 485–9 (2012). 21750395 10.3275/7860

[b44] Nadra A. D., Serrano L. & Alibés A. DNA-binding specificity prediction with FoldX. Methods in Enzymology 498, 3–18 (2011). 21601671 10.1016/B978-0-12-385120-8.00001-2

[b45] Wu D. A. & Chung B. Mutations of P45Oc21 (Steroid 21-Hydroxylase) at Cys428, Val281, and Serf” Result in Complete, Partial, or No Loss of Enzymatic Activity, Respectively. J. Clin. Invest. 88, 519–523 (1991). 1864962 10.1172/JCI115334 PMC295377

[b46] Ozturk I. C., Wei W.-L., Palaniappan L., Rubenfire M. & Killeenas A. A. Analysis of CYP21 Coding Polymorphisms in Three Ethnic Populations: Further Evidence of Nonamplifying CYP21 Alleles Among Whites. Mol. Diagnosis 5, 47–52 (2000). 10.1007/BF03262022 10837089

[b47] Asanuma A. . Molecular analysis of Japanese patients with steroid 21-hydroxylase deficiency. J. Hum. Genet. 44, 312–317 (1999). 10496074 10.1007/s100380050167

[b48] Dolzan V. . Mutational spectrum of steroid 21-hydroxylase and the genotype-phenotype association in Middle European patients with congenital adrenal hyperplasia. Eur. J. Endocrinol. 153, 99–106 (2005). 15994751 10.1530/eje.1.01944

[b49] Nikoshkov A., Lajic S., Holst M., Wedell A. & Luthman H. Synergistic effect of partially inactivating mutations in steroid 21-hydroxylase deficiency. J. Clin. Endocrinol. Metab. 82, 194–199 (1997). 8989258 10.1210/jcem.82.1.3678

[b50] Menassa R. . p.H62L, a rare mutation of the CYP21 gene identified in two forms of 21-hydroxylase deficiency. J. Clin. Endocrinol. Metab. 93, 1901–1908 (2008). 18319307 10.1210/jc.2007-2701

[b51] Tardy V. . Phenotype-genotype correlations of 13 rare CYP21A2 mutations detected in 46 patients affected with 21-hydroxylase deficiency and in one carrier. J. Clin. Endocrinol. Metab. 95, 1288–1300 (2010). 20080860 10.1210/jc.2009-1202

[b52] Sherry S. T. . dbSNP: the NCBI database of genetic variation. Nucleic Acids Res. 29, 308–311 (2001). 11125122 10.1093/nar/29.1.308 PMC29783

[b53] Sudmant P. H. . An integrated map of structural variation in 2,504 human genomes. Nature 526, 75–81 (2015). 26432246 10.1038/nature15394 PMC4617611

[b54] Sali A. & Blundell T. L. Comparative protein modelling by satisfaction of spatial restraints. J. Mol. Biol. 234, 779–815 (1993). 8254673 10.1006/jmbi.1993.1626

[b55] Tamura K., Dudley J., Nei M. & Kumar S. MEGA4: Molecular Evolutionary Genetics Analysis (MEGA) software version 4.0. Mol. Biol. Evol. 24, 1596–1599 (2007). 17488738 10.1093/molbev/msm092

[b56] Shen M.-Y. & Sali A. Statistical potential for assessment and prediction of protein structures. Protein Sci. 15, 2507–2524 (2006). 17075131 10.1110/ps.062416606 PMC2242414

[b57] Benkert P., Biasini M. & Schwede T. Toward the estimation of the absolute quality of individual protein structure models. Bioinformatics 27, 343–350 (2011). 21134891 10.1093/bioinformatics/btq662 PMC3031035

[b58] Ramachandran G. N., Ramakrishnan C. & Sasisekharan V. Stereochemistry of polypeptide chain configurations. J. Mol. Biol. 7, 95–99 (1963). 13990617 10.1016/s0022-2836(63)80023-6

[b59] Schymkowitz J. . The FoldX web server: an online force field. Nucleic Acids Res. 33, W382–388 (2005). 15980494 10.1093/nar/gki387 PMC1160148

[b60] Soardi F. C. . Inhibition of CYP21A2 enzyme activity caused by novel missense mutations identified in Brazilian and Scandinavian patients. J Clin Endocrinol Metab 93, 2416–2420 (2008). 18381579 10.1210/jc.2007-2594

[b61] Speiser P. W. . Congenital adrenal hyperplasia due to steroid 21-hydroxylase deficiency: an Endocrine Society clinical practice guideline. J Clin Endocrinol Metab 95, 4133–4160 (2010). 20823466 10.1210/jc.2009-2631 PMC2936060

