# Supplementary material for: Structure-based activity prediction of CYP21A2 stability variants: A survey of available gene variations
Source: Sci Rep. 2016 Dec 14;6:39082. doi: 10.1038/srep39082 (PMC5155424; doi:10.1038/srep39082)
Supplement: Supplementary Information [file srep39082-s1.pdf]

SUPPLEMENTARY INFORMATION

Structure-based activity prediction of CYP21A2 stability variants: A survey of available gene variations

Carlos D. Bruque [1,2], Marisol Delea [1], Cecilia S. Fernández [1], Juan V. Orza [1], Melisa Taboas [1], Noemí Buzzalino N [1], Lucía D. Espeche [1], Andrea Solarí[1], Verónica Luccerini [3], Liliana Alba [1], Alejandro D. Nadra [4 *] and Liliana Dain [1,2 *]

1. Centro Nacional de Genética Médica, ANLIS, Buenos Aires, Argentina,
2. Instituto de Biología y Medicina Experimental, CONICET, Buenos Aires, Argentina.
3. Consultorio y Laboratorio de Genética, Rosario, Argentina.
4. Departamento de Química Biológica Facultad de Ciencias Exactas y Naturales, Universidad de Buenos Aires, IQUIBICEN-CONICET, Buenos Aires, Argentina.

***** Corresponding authors. E-mail: ldain@fbmc.fcen.uba.ar; anadra@qi.fcen.uba.ar

MATERIALS AND METHODS

CYP21A2 genotyping:

Endocrine and genetic evaluations of the patients were conducted at the Division Endocrinología of the Hospital Durand and at the Centro Nacional de Genética Médica, Buenos Aires, Argentina. Patients were included following the diagnostic criteria already described [1,2].
Nucleotide numbering was performed following the guidelines of the Human Genome Variation Society [3], using M13936.1 [4] as the genomic *CYP21A2* reference sequence. All new data have been deposited in the GeneBank database. CYP21A2 Isoform 1 (NP_000491.4) has been chosen as the canonical sequence.
DNA was isolated from peripheral blood leukocytes and disease-causing mutations were analyzed by direct sequencing, as previously described [5]. Briefly, the entire coding region and the proximal promoter of the *CYP21A2* gene (416 bp 5' upstream the first ATG to 2792 bp downstream in the 3´UTR) were amplified in four gene-specific overlapping fragments and PCR products were sequenced following the Big Dye terminator sequencing protocol (Applied Biosystems, Foster City, CA, USA). *CYP21A2/CYP21A1P* deletions/duplications and large gene conversion were performed by MLPA (SALSA P050-B3 CAH MLPA Mix, MRC-Holland BV, Amsterdam, Holland). In addition, long range PCRs were performed as according to Lee H and Parajes et al. [6,7], to determine segregation of mutations in duplicated *CYP21A2* haplotypes. When available, DNA samples from parents were also analyzed.

Sequence alignments:

Similarities between CYP21 proteins were assessed using sequences retrieved from different mammalian species: *Homo sapiens* (NP_000491.4); *Pan troglodytes* (Chimpanzee, RefSeq: XP_003311237); *Macaca mulatta* (NP_001181556); *Otolemur garnettii* (ENSOGAP00000008606); *Oryctolagus cuniculus* (ENSOCUP00000006096); *Mus musculus* (NP_034125); Rattus norvegicus (NP_476442.2); *Bos taurus* (NP_001013614); *Equus caballus* (XP_001491972; *Canis familiaris* (NP_001003335). Partial fragments were aligned using MEGA 4 software [8] (http://www.megasoftware.net /mega4/index.html) using default parameters for pairwise and multiple alignments (GOP 15; GEP 6,66). Visualization was performed with GENEDOC software [9] (https://www.psc.edu/index.php/user-resources/ software/genedoc).

REFERENCES

1. Dain L.B. *et al*. Classical and nonclassical 21-hydroxylase deficiency: a molecular study of Argentine patients. *Clin Endocrinol (Oxf)* 56:239–245 (2002).
2. Pasqualini T. *et al*. Congenital adrenal hyperplasia clinical characteristics and genotype in newborn, childhood and adolescence. *Medicina (B Aires)* 67:253–61 (2007).

3. den Dunnen J.T. & Antonarakis S.E. 2002. Mutation nomenclature extensions and suggestions to describe complex mutations: A discussion. *Hum Mutat* 15:7–12 (2002).
4. White P.C., New M.I. & Dupont B. Structure of human steroid 21-hydroxylase genes. *Proc Natl Acad Sci U S A* 83:5111–5115 (1986).
5. Minutolo C., *et al*. Structure-based analysis of five novel disease-causing mutations in 21-hydroxylase-deficient patients. *PLoS One* 6:e15899 (2011).
6. Lee HH. The chimeric CYP21P/CYP21 gene and 21-hydroxylase deficiency. J *Hum Genet* 49:65–72 (2004).
7. Parajes S., Quinteiro C., Domínguez F. & Loidi L. High frequency of copy number variations and sequence variants at *CYP21A2* locus: implication for the genetic diagnosis of 21-hydroxylase deficiency. *PLoS One* 3:e2138 (2008).
8. Tamura K., Dudley J., Nei M., &Kumar S. MEGA4: Molecular Evolutionary Genetics Analysis (MEGA) software version 4.0. *Mol Biol Evol* 24:1596–1599 (2007).
9. Nicholas K.B., Nicholas H.B.Jr. & Deerfield D.W. GeneDoc: analysis and visualization of genetic variation. *EMBnet News* **4**:14 (1997).

Table S1: Aminoacidic variants reported in CYP21A2 human protein, *in vitro* activities and proposed impairment mechanism in protein dysfunction and/or location in the structure.

Variant	In vitro residual activity ± SD		Proposed effect (Bovine model/Human crystal)	ΔΔG ± SD		References
	17- OHP	P		Bovine model	Human crystal structure	
**p.M1I**	ND	ND	Start codon	NA	NA	1
**p.M1L**	ND	ND	Start codon	NA	NA	2
**p.M1V**	ND	ND	Start codon	NA	NA	3
**p.M1T**	ND	ND	Start codon	NA	NA	4
**p.L9_10dup**	96.5 ± 7.6	94 ± ND	Dup	NA	NA	5
**p.P10S**	ND	ND	PTR	NA	NA	rs764653902:C>T
**p.A13S**	ND	ND	PTR	NA	NA	rs764636694:G>T
**p.A15T**	95 ± 15	101 ± 9	PTR	NA	NA	6
**p.R16C**	ND	ND	PTR	NA	NA	rs757608533:C>T
**p.W19***	ND	ND	Nonsense	NA	NA	7, 8
**p.W19C**	ND	ND	PTR	NA	NA	rs746097144:C>G
**p.W21***	ND	ND	Nonsense	NA	NA	rs756302021:G>A
**p.W22***	ND	ND	Nonsense	NA	NA	9, 10
**p.W22fs**	ND	ND	Indel	NA	NA	11
**p.R25W**	ND	ND	PTR	NA	NA	rs749346869:C>T
**p.R25Q**	ND	ND	PTR	NA	NA	rs771942449:G>A
**p.S26G**	ND	ND	PTR	NA	NA	rs773259857:A>G
**p.H28Y**	ND	ND	PTR	NA	NA	rs770843305:C>T
**p.H28fs**	ND	ND	Indel	NA	NA	12, 13
**p.P30L**	34.1 ± 18.5	38.8 ± 22.2	Stability	2.4 ± 1.1	5.48 ± 1.14	14
**p.P30Q**	0.2 ± 0.2	0 ± 0	Stability	4.7 ± 0.7	6.85 ± 0.51	5
**p.P34L**	ND	ND	Stability	0.75 ± 0.03	0.50 ± 0.04	rs200648381:C>T
**p.H38L**	ND	ND	Stability	-1.64 ± 0.1	-0.32 ± 0.08	15
**p.P42fs**	ND	ND	Indel	NA	NA	16
**p.D43N**	ND	ND	Stability	-0.16 ± 0.02	0.09 ± 0.06	rs762507423:G>A
**p.D43G**	ND	ND	Stability	-0.41 ± 0.10	0.87 ± 0.08	rs764569922:A>G
**p.P45L**	105 ± 10.6	ND	Stability	3.72 ± 0.5	3.56 ± 0.46	17
**p.I46fs**	ND	ND	Indel	NA	NA	18
**p.Y47C**	ND	ND	Stability	-1.64 ± 0.6	2.03 ± 0.05	19
**p.L48***	ND	ND	Nonsense	NA	NA	20
**p.L48fs**	ND	ND	Indel	NA	NA	21
**p.K54***	ND	ND	Nonsense	NA	NA	22
**p.G56R**	0.7 ± ~0.2	1.4 ± ~0.5	Stability	3.2 ± 0.2	2.97 ± 0.12	23
**p.Y59N**	ND	ND	Stability	2.54 ± 0.1	5.55 ± 0.01	24
**p.H62L**	44.5 ± ~28.0	20.7 ± ~5	H-L	NA	NA	23
**p.G64E**	No activity	No activity	H-L	NA	N	25

Variant	In vitro residual activity ± SD		Proposed effect (Bovine model/Human crystal)	ΔΔG ± SD		References
	17- OHP	P		Bovine model	Human crystal structure	
**p.Q66K**	ND	ND	H-L	NA	NA	rs749374064:C>A
**p.V69L**	ND	ND	Stability	1.47 ± 0.79	-1.10 ± 0.50	26
**p.K74***	ND	ND	Nonsense	NA	NA	27
**p.R75K**	ND	ND	Stability	-0.45 ± 0.09	0.26 ± 0.09	rs368330593:G>A
**p.I77T**	3 ± 2	5 ± 3	H-L	NA	NA	28
**p.E78D**	ND	ND	Stability	1.92 ± 0.17	1.09 ± 0.02	rs762330375:G>T
**p.D87fs**	ND	ND	Indel	NA	NA	rs750910646:InsA A
**p.D87N**	ND	ND	Stability	-0.02 ± 0.55	0.28 ± 0.05	rs767973196:G>A
**p.D87G**	ND	ND	Stability	-0.32 ± 0.14	0.82 ± 0.17	rs750793252:A>G
**p.G90V**	~0 ± ND	~0 ± ND	H-L	NA	NA	27
**p.R91G**	ND	ND	H-L	NA	NA	26
**p.R91***	ND	ND	Nonsense	NA	NA	29
**p.L95P**	ND	ND	H-L$^{\&}$	NA	NA	rs761079139:T>C
**p.Y97N**	ND	ND	H-L$^{\&}$	NA	NA	rs755085518:T>A
**p.Y97***	ND	ND	Nonsense	NA	NA	30
**p.K102R**	119.7 ± 22.5	ND	Stability	0.8 ± 0.2	0.30 ± 0.25	17
**p.P105L**	62 ± 9	64 ± 12	Stability	-0.6 ± 0.4	1.90 ± 0.14	31
**p.P105T**	ND	ND	Stability	1.89 ± 0.26	1.67 ± 0.01	rs531645802:C>A
**p.D106N**	ND	ND	Stability	-0.85 ± 0.09	-3.95 ± 0.72	rs774531624:G>A
**p.L107R**	0.4± ~0.1	0.3 ± ~0.1	H-L	NA	NA	23
**p.S108P**	ND	ND	H-L	NA	NA	rs761777118:T>C
**p.G110fs**	ND	ND	Indel	NA	NA	32
**p.S113Y**	ND	ND	Stability	0.79 ± 0.19	0.81 ± 0.04	28
**p.S113F**	ND	ND	Stability	1.05 ± 0.42	0.49 ± 0.18	33
**p.H119R**	31.6 ± 8	32.5 ± 7	H-L	NA	NA	34
**p.K120Q**	ND	ND	H-L	NA	NA	rs267606757:A>C
**p.K121Q**	14 ± 5	19.5 ± 4	POR	NA	NA	35
**p.L122P**	1.42 ± 2.13	-1.86 ± 5.19	Stability	4.95 ± 0.74	4.65 ± 0.49	36
**p.T123I**	ND	ND	Stability	-1.88 ± 0.45	-3.13 ± 0.06	rs566065375:C>T
**p.R124H**	ND	ND	POR	NA	NA	1
**p.R124C**	ND	16 ± 0.6	POR	NA	NA	37
**p.L129P**	ND	ND	H-L$^{\&}$	NA	NA	38
**p.I131M**	ND	ND	Stability	0.02 ± 0.02	0.53 ± 0.39	rs746395253:C>G
**p.R132C**	35.40± 7.4	15.5 ± 2.7 0	POR	NA	NA	39
**p.D133Y**	ND	ND	Stability	0.14 ± 0.01	1.75 ± 0.03	rs776029298:G>T
**p.E136***	ND	ND	Nonsense	NA	NA	rs749639252:G>T
**p.E136V**	ND	ND	Stability	0.65 ± 0.04	0.07 ± 0.17	rs768973843:A>T
**p.V139E**	0.7 ± 1.3	0.5 ± 0.6	Stability	3.9 ± 0.86	7.43 ± 0.44	40
**p.E140K**	11.30 ± 2.4	ND	POR	NA	NA	17
**p.E140G**	ND	ND	POR	NA	NA	rs762017598:A>G

Variant	In vitro residual activity ± SD		Proposed effect (Bovine model/Human crystal)	ΔΔG ± SD		References
	17- OHP	P		Bovine model	Human crystal structure	
**p.Q141***	ND	ND	Nonsense	NA	NA	37
**p.L142P**	0.4 ± ~0.2	0.4 ± ~0.1	Stability	7.2 ± 0.1	4.96 ± 0.25	23
**p.Q144P**	ND	ND	Stability	0.61 ± 0.21	2.58 ± 0.52	26
**p.C147R**	4.3 ± 0.9	3.6 ± 1.8	Stability	1.47 ± 0.51	1.14 ± 0.42	40
**p.R149P**	23.4 ± 1.7	16.9 ± 2	Stability	1.99 ± 0.17	3.81 ± 0.20	41
**p.R149C**	35.8 ± 14.6	47.3 ± 12.9	Stability	0.2 ± 0.22	0.78 ± 0.05	39
**p.R149S**	ND	ND	Stability	0.02 ± 0.18	0.00 ± 0.03	rs577450124:C>A
**p.R149H**	ND	ND	Stability	0.37 ± 0.19	0.36 ± 0.05	rs760710835:G>A
**p.M150R**	17.66 ± 1.87	4.57 ± 1.87	Stability	3.61 ± 0.93	2.88 ± 0.15	36
**p.M150V**	ND	ND	Stability	0.53 ± 0.14	1.94 ± 0.21	rs769769128:A>G
**p.Q153***	ND	ND	Nonsense	NA	NA	26
**p.G155S**	ND	ND	Stability	0.77 ± 0.01	1.97 ± 0.01	rs541292262:G>A
**p.A159T**	126.6 ± 29.9	ND	Stability	0.39 ± 0.01	0.75 ± 0.01	17
**p.I160fs**	ND	ND	Indel	NA	NA	42
**p.E161***	0.29 ± 0.11	0.18 ± 0	Nonsense	NA	NA	36
**p.E163A**	ND	ND	Stability	2.16 ± 0.45	0.30 ± 0.03	rs767249456:A>C
**p.F164V**	ND	ND	Stability	3.13 ± 0.05	4.48 ± 0.01	rs755674550:T>G
**p.F164S**	ND	ND	Stability	1.37 ± 0.08	5.83 ± 0.03	26
**p.S165P**	ND	ND	Stability	4.94 ± 0.26	3.17 ± 0.36	38
**p.L166P**	0.30 ± 0.06	0.4 ± 0.6	Stability	18.17 ± 0.04	5.90 ± 0.04	43
**p.L167P**	0.7 ± ND	0.4 ± ND	Stability	3.5 ± 0.5	8.15 ± 0.21	14
**p.T168N**	ND	ND	Stability	3.41 ± 0.27	1.98 ± 0.35	44
**p.C169R**	0.10 ± 0.02	0 ± 2	Disulphide bridge	NA	NA	45
**p.C169fs**	ND	ND	Indel	NA	NA	46
**p.C169***	ND	ND	Nonsense	NA	NA	44
**p.S170L**	ND	ND	Stability	-2.60 ± 0.02	-2.81 ± 0.54	rs754305318:G>T
**p.S170fs**	ND	ND	Indel	NA	NA	47
**p.I171N**	0.7 ± 0.3	0.60 ± 0.03	H-L	NA	NA	48
**p.I172N**	4.3 ± 1.7	4.4 ± 1.8	H-L	NA	NA	14
**p.L175H**	ND	ND	Stability	3.22 ± 0.72	-0.47 ± 3.88	rs779166970:T>A
**p.T176N**	ND	ND	Stability	-0.72 ± 0.90	2.39 ± 0.15	rs748501160:C>A
**p.G178A**	~19 ± ND	~0 ± ND	Stability	1.35 ± 0.01	4.01 ± 0.13	27
**p.G178R**	0.4 ± 0.5	0 ± 0.6	Stability	2.58 ± 0.2	5.21 ± 0.48	45
**p.D183E**	100 ± ND	100 ± ND	Stability	0.1 ± 0.03	-0.36 ± 0.04	49
**p.D183fs**	ND	ND	Indel	NA	NA	50
**p.D184N**	ND	ND	Stability	-0.97 ± 0.07	-0.09 ± 0.01	rs745933819:G>A
**p.Y190N**	ND	ND	Stability	-1.95 ± 1.06	4.70 ± 0.06	rs768524764:T>A
**p.Y191H**	37.1 ± 7	25.8 ± 9	Stability	1.9 ± 0.03	2.84 ± 0.01	51
**p.I194N**	33.2 ± 9	46.7 ± 10	Stability	1.2 ± 0.2	1.91 ± 0.02	34

Variant	In vitro residual activity ± SD		Proposed effect (Bovine model/Human crystal)	ΔΔG ± SD		References
	17- OHP	P		Bovine model	Human crystal structure	
**p.E196del**	ND	ND	Del	NA	NA	52
**p.V197L**	ND	ND	H-L	NA	NA	rs565063947:G>T
**p.V197M**	ND	ND	H-L	NA	NA	rs565063947:G>A
**p.L198F**	ND	ND	H-L	NA	NA	rs143240527:A>T
**p.T200A**	ND	ND	Stability	0.25 ± 0.09	-0.01 ± 0.01	rs760425681:A>G
**p.S202G**	ND	ND	Stability	0.57 ± 0.03	-0.24 ± 0.03	rs372964292:A>G
**p.H203Y**	ND	ND	Stability	-0.02 ± 0.01	-0.12 ± 0.08	rs776040958:C>T
**p.Q207***	ND	ND	Nonsense	NA	NA	rs565829460:C>T
**p.V211M**	99.5 ± 32.4	ND	Stability	-0.89 ± 0.09	0.49 ± 0.38	17
**p.V211L**	ND	ND	Stability	-0.91 ± 0.08	0.53 ± 0.18	53
**p.I212T**	ND	ND	Stability	1.51 ± 0.09	2.34 ± 0.12	rs764468228:T>C
**p.P213fs**	ND	ND	Indel	NA	NA	1
**p.P219L**	ND	ND	Stability	0.67 ± 0.21	0.70 ± 0.06	rs770752895:C>T
**p.N220S**	ND	ND	Stability	0.00 ± 0.05	0.58 ± 0.02	rs780780640:A>G
**p.N220fs**	ND	ND	Indel	NA	NA	54
**p.G222C**	ND	ND	Stability	1.75 ± 0.05	2.95 ± 0.45	rs769293380:G>T
**p.L223P**	ND	ND	Stability	-0.68 ± 0.30	0.03 ± 0.06	rs775023782:T>C
**p.R224W**	51.9 ± 9	45.6 ± 8	POR	NA	NA	55
**p.R225fs**	ND	ND	Indel	NA	NA	29
**p.K227fs**	ND	ND	Indel	NA	NA	56
**p.Q228***	ND	ND	Nonsense	NA	NA	57
**p.I230T**	63.1 ± 22.3	70.6 ± 17	Stability/H-L	0.6 ± 0.27	NA	14
**p.R233K**	15 ± ND	8.1 ± ND	H-L	NA	NA	14
**p.R233G**	8 ± 2	2 ± 1	H-L	NA	NA	58
**p.D234E**	ND	ND	Stability	-0.43 ± 0.21	1.31 ± 0.04	rs10947229:T>G
**p.D234N**	ND	ND	Stability	-0.51 ± 0.47	0.50 ± 0.01	rs774835577:G>A
**p.I236V**	ND	ND	ClEx6*	NA	NA	rs750703275:A>G
**p.I236N**	1 ± 0.1	2.4 ± 1.4	ClEx6*	NA	NA	59
**p.I237_M240 delinsNEEL**	ND	ND	Indel	NA	NA	rs786204728:delTCGTGGAGATinsACGAGGAGAA
**p.V237M**	ND	ND	ClEx6*	NA	NA	rs148288899:G>A
**p.V237E**	0 ± 0	0.1 ± 0.3	ClEx6*	NA	NA	59
**p.E238K**	ND	ND	Stability	-0.33 ± 0.55	-0.41 ± 0.03	60
**p.M239K**	95.4 ± 24.7	97.7 ± 7.7	ClEx6*	NA	NA	59
**p.R242S**	ND	ND	Stability	0.65 ± 0.28	0.45 ± 0.05	rs150496227:G>C

**Variant**	**In vitro residual activity ± SD**		**Proposed effect (Bovine model/Human crystal)**	**ΔΔG ± SD**		**References**
	17- OHP	P		Bovine model	Human crystal structure	
**p.H244R**	ND	ND	Stability	5.49 ± 1.30	0.15 ± 0.34	rs553299378:A>G
**p.E246fs**	ND	ND	Indel	NA	NA	61
**p.S247R**	ND	ND	Stability	-1.34 ± 0.06	0.20 ± 0.37	rs772680196:C>G
**p.V249A**	ND	ND	Stability	0.45 ± 0.01	0.51 ± 0.03	62
**p.G251S**	ND	ND	Stability	1.62 ± 0.01	3.06 ± 0.01	rs182942340:G>A
**p.W253R**	ND	ND	Stability	-0.80 ±0.34	1.11 ± 0.42	rs759857632:T>C
**p.R254K**	ND	ND	Stability	-0.01 ± 0.11	1.14 ± 0.05	rs559386220:G>A
**p.D255E**	ND	ND	Stability	6.36 ± 0.91	5.84 ± 0.14	rs775688953:C>A
**p.M256T**	ND	ND	Stability	1.54 ± 0.26	5.34 ± 0.02	rs762929624:T>C
**p.M260V**	ND	ND	Stability	0.67 ± 0.03	2.63 ± 0.02	rs767558605:A>G
**p.L261P**	ND	ND	Stability	8.95 ± 0.11	6.47 ± 0.05	63
**p.Q262***	ND	ND	Nonsense	NA	NA	25
**p.Q262fs**	ND	ND	Indel	NA	NA	64
**p.A265V**	92.0 ± 1.40	100 ± 4.3	Stability	0.47 ± 0.01	0.17 ± 0.21	65
**p.A265S**	90 ± 9	104 ± 15	Stability	-0.03 ± 0.01	-0.45 ± 0.23	58
**p.P267L**	ND	ND	Stability/PTR	-1.02 ± 0.24	NA	rs142028935:C>T
**p.S268C**	93 ± 7	ND	Stability/PTR	0.3 ± 0.04	NA	66
**p.S268M**	107 ± 2	ND	Stability/PTR	0.4 ± 0.18	NA	66
**p.S268T**	103 ± 15	ND	Stability/PTR	0.74 ± 0.03	NA	66
**p.S268G**	ND	ND	Stability/PTR	-0.85 ± 0.03	NA	rs752306014:A>G
**p.S268fs**	ND	ND	Indel	-0.53 ± 0.18	NA	rs760900241:delC
**p.S273Y**	ND	ND	Stability/PTR	0.30 ± 0.01	NA	rs78884659:C>A
**p.L276S**	ND	ND	Stability	3.25 ± 0.08	4.19 ± 0.09	rs747482395:C>A
**p.V281G**	3.9 ± 1.7	3.9 ± 2	H-L**[&]	NA	NA	67
**p.V281I**	45.0 ± 5	ND	H-L**[&]	NA	NA	66
**p.V281L**	65.6 ± 10.9	63.4 ± 8.7	H-L**[&]	NA	NA	14
**p.V281T**	10.0 ± 8	ND	H-L**[&]	NA	NA	66
**p.H282N**	1.6 ± 6	2.7 ± 5	H-L[&]	NA	NA	51
**p.M283L**	ND	ND	Stability	-0.30 ± 0.13	-0.15 ± 0.09	68
**p.M283V**	16.2 ± 9.3	19 ± 6.8	Stability	1.08 ± 0.23	1.55 ± 0.20	39
**p.A284P**	ND	ND	Stability	6.11 ± 1.11	8.43 ± 0.06	rs775570889:G>C
**p.A285T**	ND	ND	Stability	1.51 ± 0.46	1.28 ± 0.70	rs570785206:G>A
**p.L288F**	ND	ND	H-L	NA	NA	rs768635138:C>T
**p.G291C**	~0 ± ND	~0 ± ND	H-L	NA	NA	27
**p.G291R**	0.5 ± 0.7	0.7 ± 0.2	H-L	NA	NA	40
**p.G291S**	0.8 ± 0.4	0.8 ± 0.4	H-L	NA	NA	50
**p.G292D**	0.5 ± 0.2	0.7 ± 0.4	H-L	NA	NA	14
**p.G292S**	ND	ND	H-L	NA	NA	rs151344501:G>A
**p.E294K**	ND	ND	H-L	NA	NA	rs148622274:G>A
**p.T295N**	5.0 ± 1.6	0.8 ± 0.4	H-L	NA	NA	40

Variant	In vitro residual activity ± SD		Proposed effect (Bovine model/Human crystal)	ΔΔG ± SD		References
	17- OHP	P		Bovine model	Human crystal structure	
**p.T295A**	ND	ND	H-L	NA	NA	rs759308952:A>G
**p.L300F**	9.5 ± 6.4	4.4 ± 2.5	H-L	NA	NA	67
**p.L300P**	ND	ND	H-L	NA	NA	rs752295510:T>C
**p.S301Y**	ND	ND	Stability	12.31 ± 3.35	22.42 ± 0.39	50
**p.W302R**	0.1 ± 0.2	0 ± 0.5	Stability	4.2 ± 0.43	7.62 ± 0.99	45
**p.W302S**	3.0 ± 0.3	3 ± 0.5	Protein degradation	NA	NA	65
**p.W302***	ND	ND	Nonsense	NA	NA	69
**p.V304M**	46 ± ~18	26 ± ~10	Stability	1.01 ± 1.19	-0.50 ± 0.29	70
**p.V304E**	ND	ND	Stability	2.44 ± 0.28	3.48 ± 0.05	26
**p.V305D**	ND	ND	Stability	0.99 ± 0.70	3.41 ± 0.04	33
**p.V305A**	ND	ND	Stability	-1.47 ± 0.02	2.37 ± 0.05	rs568758408:T>C
**p.F306V**	ND	ND	Stability	1.87 ± 0.16	5.89 ± 0.11	33
**p.L307fs**	ND	ND	Indel	NA	NA	32, 57
**p.L307V**	ND	ND	Stability	2.80 ± 0.12	2.03 ± 0.10	29 New et al.,2013
**p.L308F**	0.2 ± 0.3	0.1 ± 0.3	Stability	5.75 ± 0.23	3.78 ± 0.64	40
**p.H310N**	ND	ND	Stability	-0.54 ± 0.65	0.96 ± 0.02	rs770059546:C>A
**p.P311S**	ND	ND	Stability	0.76 ± 0.07	2.32 ± 0.01	rs536088585:C>T
**p.I313L**	ND	ND	Stability	1.03 ± 0.22	-0.53 ± 0.20	rs376415981:A>C
**p.Q315***	ND	ND	Nonsense	NA	NA	71
**p.R316***	ND	ND	Nonsense	NA	NA	72
**p.R316L**	ND	ND	Stability	0.03 ± 0.08	-0.28 ± 0.04	29
**p.L317M**	ND	ND	Stability	-0.80 ± 0.07	-0.02 ± 0.37	73
**p.L317V**	ND	ND	Stability	2.42 ± 0.11	3.04 ± 0.06	74
**p.Q318***	ND	ND	Nonsense	NA	NA	75
**p.E320K**	4.6 ± 1.8	4.5 ± 2.6	POR	NA	NA	14
**p.L321P**	ND	ND	Stability	9.84 ± 0.20	5.16 ± 0.04	33
**p.D322H**	ND	ND	Stability	-0.52 ± 0.40	-1.30 ±0.07	rs142058202:G>C
**p.D322G**	18.0 ± 1.2	27 ± 4.7	Protein degradation	NA	NA	65
**p.E324K**	ND	ND	Stability	-0.99 ± 0.08	-1.71 ± 0.1	rs150804717:G>A
**p.E324G**	ND	ND	Stability	0.16 ± 0.16	-0.06 ± 0.19	rs745826667:A>G
**p.G326S**	ND	ND	Stability/PTR	0.34 ± 0.32	NA	rs769730855:G>A
**p.A329V**	ND	ND	Stability/PTR	0.29 ± 0.02	NA	rs774151904:C>T
**p.S330fs**	ND	ND	Indel	NA	NA	72
**p.S331G**	ND	ND	Stability/PTR	-0.77 ± 0.01	NA	rs767161968:A>G
**p.R333W**	ND	ND	Stability	0.44 ± 0.22	0.50 ± 0.52	rs749917058:C>T
**p.R333Q**	ND	ND	Stability	-0.08 ± 0.07	-0.18 ± 0.32	rs756621561:G>A
**p.V334fs**	ND	ND	Indel	NA	NA	37
**p.Y336***	ND	ND	Nonsense	NA	NA	76
**p.Y336H**	ND	ND	Stability	0.02 ± 0.20	2.12 ± 0.36	rs754209599:T>C

**Variant**	**In vitro residual activity ± SD**		**Proposed effect (Bovine model/Human crystal)**	**ΔΔG ± SD**		**References**
	17- OHP	P		Bovine model	Human crystal structure	
**p.R339H**	ND	ND	POR	NA	NA	77.
**p.R339C**	ND	ND	POR	NA	NA	rs548446169:C>T
**p.R339***	ND	ND	Nonsense	NA	NA	29
**p.R341P**	0.7 ± 0.3	0.7 ± 0.2	POR	NA	NA	48
**p.R341W**	5 ± 0.4	4 ± 3	POR	NA	NA	58
**p.R341Q**	ND	ND	POR	NA	NA	rs747079101:G>A
**p.T348S**	ND	ND	Stability	1.27 ± 0.01	0.84 ± 0.01	rs370908729:C>G
**p.I349M**	ND	ND	Stability	-1.23 ± 0.05	-0.39 ± 0.17	rs144104274:C>G
**p.A350T**	ND	ND	Stability	0.77 ± 0.32	0.51 ± 0.05	rs768586475:G>A
**p.A350V**	ND	ND	Stability	1.60 ± 0.27	0.13 ±0.01	rs774242376:C>T
**p.E351D**	3.4 ± 0.8	3.6 ± 0.5	Meander and the ERR-Triad	NA	NA	78
**p.E351I**	0.9 ± 0.7	0.8 ± 0.7	Meander and the ERR-Triad	NA	NA	78
**p.E351K**	1.1 ± 0.5	1.2 ± 0.3	Meander and the ERR-Triad	NA	NA	78
**p.E351V**	ND	ND	Meander and the ERR-Triad	NA	NA	79
**p.L353R**	ND	ND	H-L	NA	NA	80
**p.R354C**	ND	ND	Meander and the ERR-Triad	NA	NA	81
**p.R354H**	~10 ± ~5	~0 ± ND	Meander and the ERR-Triad	NA	NA	27
**p.R356P**	0.15 ± 0.30	0.15 ± 0.30	POR	NA	NA	82
**p.R356Q**	0.65 ± 0.44	1.1 ± 0.94	POR	NA	NA	82
**p.R356W**	No activity	No activity	POR	NA	NA	83
**p.V358I**	ND	ND	H-L	NA	NA	rs373579128:G>A
**p.A362V**	No activity	No activity	H-L	NA	NA	25
**p.L363W**	ND	ND	H-L	NA	NA	84
**p.H365Y**	ND	ND	H-L	NA	NA	16
**p.R366C**	37 ± 7	28 ± 4	H-L[&]	NA	NA	58
**p.R366H**	ND	ND	H-L [&]	NA	NA	33
**p.R366S**	ND	ND	H-L[&]	NA	NA	rs758658540:C>A
**p.R366G**	ND	ND	H-L [&]	NA	NA	rs758658540:C>G
**p.R369Q**	ND	ND	POR	NA	NA	rs376421504:G>A
**p.R369W**	45.8 ± 1.8	48.5 ± 17.1	POR	0.67 ± 0.24	1.96 ± 0.13	14
**p.G375S**	1.6 ± ~0.8	0.7 ± ~0.7	Stability	1.32 ± 0.07	2.46 ± 0.01	70
**p.Y376***	ND	ND	Nonsense	NA	NA	50
**p.D377N**	ND	ND	Stability	0.17 ± 0.02	0.23 ± 0.02	rs528524868:G>A
**p.E380D**	30 ± ND	ND	POR	NA	NA	85

Variant	In vitro residual activity ± SD		Proposed effect (Bovine model/Human crystal)	ΔΔG ± SD		References
	17- OHP	P		Bovine model	Human crystal structure	
**p.G381S**	ND	ND	Stability	3.51 ± 0.01	3.41± 0.15	33
**p.P386L**	ND	ND	H-L	0.13 ± 0.02	1.69 ± 1.19	33
**p.P386R**	ND	ND	H-L	0.21 ± 0.11	6.02 ± 1.32	44
**p.N387K**	ND	ND	Stability	7.55 ± 0.78	10.80 ± 1.45	86 9
**p.L388R**	1.1 ± 0.6	ND	H-L	NA	NA	17
**p.L388F**	ND	ND	H-L	NA	NA	rs746692392:C>T
**p.A391T**	38.7 ± 9.5	22.9 ± 4.7	Stability	1.56 ± 0.09	1.92 ± 0.51	43
**p.H392Q**	ND	ND	Stability	1.11 ± 0.04	4.29 ± 0.41	rs745358717:C>G
**p.T396M**	ND	ND	Stability	0.18 ± 0.14	0.95 ± 0.19	rs568795145:C>T
**p.V397fs**	ND	ND	Indel	NA	NA	72
**p.V397I**	ND	ND	Stability	-0.24 ± 0.59	-0.43 ± 0.08	rs763395640:G>A
**p.E399G**	ND	ND	Stability	0.08 ± 0.05	1.71 ± 0.02	rs764542690:A>G
**p.H402R**	ND	ND	Stability	-0.50 ± 0.06	-0.30 ± 0.18	rs774540237:A>G
**p.F404S**	ND	ND	Stability	5.34 ± 0.14	5.60 ± 0.10	20
**p.F404L**	ND	ND	Stability	2.51 ± 0.66	1.94 ± 0.13	29
**p.W405***	ND	ND	Nonsense	NA	NA	87
**p.D407N**	72.7 ± 7	73.6 ± 10	Stability	0.14 ± 0.01	0.88 ± 0.01	55
**p.R408L**	ND	ND	Meander and the ERR-Triad	NA	NA	88
**p.R408C**	1.3 ± ~0.5	0.6 ± ~0.3	Meander and the ERR-Triad	NA	NA	23
**p.R408H**	ND	ND	Meander and the ERR-Triad	NA	NA	64
**p.L419P**	ND	ND	Stability	-0.40 ± 0.02	2.03 ± 0.17	rs761006767:T>C
**p.G422S**	ND	ND	H-L	NA	NA	rs776897591:G>A
**p.G424S**	1.6 ± 0.4	2 ± 0.6	H-L[&]	NA	NA	14
**p.G424fs**	ND	ND	Indel	NA	NA	64
**p.G424_R426 del**	ND	ND	Del	NA	NA	29
**p.R426C**	0 ± 0.5	0 ± 0.6	H-L	NA	NA	45
**p.R426H**	0.5 ± 0.6	0.4 ± 0.2	H-L	NA	NA	48
**p.R426P**	ND	ND	H-L	NA	NA	64
**p.C428R**	ND	ND	H-L	NA	NA	26
**p.E431K**	26.2 ± 3.8	24.2 ± 7.4	POR	NA	NA	39
**p.P432L**	ND	ND	H-L	NA	NA	89
**p.L433P**	ND	ND	H-L	NA	NA	29
**p.A434V**	14.0 ± 2	12 ± 6	H-L	NA	NA	28
**p.R435C**	ND	6.5 ± 0.9	POR	NA	NA	37
**p.V441L**	ND	ND	Stability	0.02 ± 0.44	0.07 ± 0.67	rs750190235:G>C
**p.T443I**	ND	ND	Stability	1.81 ± 0.58	-1.26 ± 0.04	rs755816115:C>T
**p.R444***	ND	ND	Nonsense	NA	NA	18, 56

Variant	In vitro residual activity ± SD		Proposed effect (Bovine model/Human crystal)	ΔΔG ± SD		References
	17- OHP	P		Bovine model	Human crystal structure	
**p.R444P**	ND	ND	POR	NA	NA	33
**p.L446P**	0.5 ± 0.60	0 ± 0.10	Stability	4.75 ± 0.4	6.93 ± 0.26	48
**p.T450P**	ND	ND	Stability	9.35 ± 0.26	4.66 ± 0.06	20
**p.P453S**	36.0 ± 5	44 ± 3	Stability	2.18 ± 0.01	2.35 ± 0.01	35
**p.P459H**	ND	6.8 ± 2.1	Stability	4.44 ± 0.08	3.42 ± 0.15	90
**p.P459S**	ND	ND	Stability	2.68 ± 0.01	2.95 ± 0.03	44
**p.P459L**	ND	ND	Stability	1.29 ± 0.23	2.04 ± 0.16	26
**p.S460_P465 del**	ND	ND	Del	NA	NA	8
**p.P463L**	2.6 ± 0.8	3 ± 0.5	PTR	NA	NA	56
**p.L464fs**	ND	ND	Indel	NA	NA	26
**p.P465***	ND	ND	Nonsense	NA	NA	29
**p.P465S**	ND	ND	Stability	1.54 ± 0.02	0.26 ± 0.01	rs748620874:C>T
**p.V469L**	ND	ND	H-L/Stability	NA	-1.37 ± 0.15	rs772588285:G>A
**p.G470fs**	ND	ND	Indel	NA	NA	91
**p.M473I**	85 ± 7	66 ± 12	Stability	0.28 ± 0.29	1.23 ± 0.08	58
**p.M473_R479 dup**	ND	ND	Dup	NA	NA	18
**p.P475fs**	ND	ND	Indel	NA	NA	92
**p.R479L**	75.5 ± 15.70	79.6 ± 12	PTR/Stability	NA	0.00 ± 0.22	43
**p.R479W**	ND	ND	PTR/Stability	NA	2.99 ± 0.18	rs778319095:C>T
**p.R479Q**	ND	ND	PTR/Stability	NA	0.95 ± 0.23	rs184649564:G>A
**p.Q481P**	ND	ND	PTR	NA	NA	93
**p.Q481***	2.98 ± 4.08	0.07 ± 0.35	Nonsense	NA	NA	35
**p.P482S**	72.0 ± 9	70 ± 12	PTR	NA	NA	6
**p.R483P**	68 ± 18	46 ± 8	PTR	NA	NA	50
**p.R483W**	ND	2.9 ± 1.5	PTR	NA	NA	90
**p.R483Q**	1.1 ± 0.7	3.8 ± 1.9	PTR	NA	NA	43
**p.R483fs**	ND	ND	Indel	NA	NA	7, 94
**p.M485fs**	ND	ND	Indel	NA	NA	rs749280425:delG
**p.M485L**	ND	ND	PTR	NA	NA	rs767421046:A>T
**p.A487T**	ND	ND	PTR	NA	NA	rs750280177:G>A
**p.S489T**	ND	ND	PTR	NA	NA	rs760461440:G>C
**p.P490L**	ND	ND	PTR	NA	NA	rs766099537:C>T
**p.G491S**	ND	ND	PTR	NA	NA	rs753450017:G>A
**p.G491D**	ND	ND	PTR	NA	NA	rs754606900:G>A
**p.G491V**	ND	ND	PTR	NA	NA	rs754606900:G>T
**p.Q492H**	ND	ND	PTR	NA	NA	rs574408366:G>C
**p.N493S**	ND	ND	PTR	NA	NA	95
**p.N493T**	ND	ND	PTR	NA	NA	rs397515529:A>C
**p.Q494fs**	ND	ND	Indel	NA	NA	60

All the mutations and SNPs reported up to date were retrieved. I*n vitro* enzymatic activities are those reported in the literature and are expressed in percentage relative to the wild type protein considered as 100%. ΔΔG was calculated only for those residues presumed to affect protein stability and is expressed in kcal/mol relative to free energy of the wild type protein. *: The p.I236N, p.V237E and p.M239K residues belong to the Cluster of Exon 6 mutation. They are located in a helix suggested to be important for initial substrate recognition, although they are not near the inner substrate-binding pocket itself [59]. **: Wu and Chung [66], showed that mutations in residue 281 impair heme incorporation to the enzyme. &: These residues have been mapped to a substrate recognition site by Arendse et. al.[96]. 17-OHP: 17-hydroxyprogesterone. P: Progesterone. POR: P450 oxidoreductase interaction; ND: Not determined; NA. Not Applicable, PTR: Poor Template Resolution: H-L: Heme or Ligand Interaction; ClEx6: Cluster Exón 6.; Dup: Duplication; Del: Deletion.

REFERENCES

1. Usui, T. *et al.* Three novel mutations in Japanese patients with 21-hydroxylase deficiency. *Horm. Res.* **61,** 126–132 (2004).
2. Tardy, V. T. V & Morel, Y. Gene symbol: CYP21A2. *Hum. Genet.* **121,** 294 (2007).
3. Tardy, V. T. V & Morel, Y. Gene symbol: CYP21A2. *Hum. Genet.* **121,** 293–294 (2007).
4. Toraman, B. *et al.* Investigation of CYP21A2 mutations in Turkish patients with 21-hydroxylase deficiency and a novel founder mutation. *Gene* **513,** 202–208 (2013).
5. Lajić, S., Nikoshkov, A., Holst, M. & Wedell, A. Effects of missense mutations and deletions on membrane anchoring and enzyme function of human steroid 21-hydroxylase (P450c21). *Biochem. Biophys. Res. Commun.* **257,** 384–390 (1999).
6. Barbaro, M. *et al.* Functional analysis of two recurrent amino acid substitutions in the CYP21 gene from Italian patients with congenital adrenal hyperplasia. *J. Clin. Endocrinol. Metab.* **89,** 2402–2407 (2004).
7. Kharrat, M. *et al.* Molecular Genetic Analysis of Tunisian Patients with a Classic Form of 21-Hydroxylase Deficiency: Identification of Four Novel Mutations and High Prevalence of Q318X Mutation. *J. Clin. Endocrinol. Metab.* **89,** 368–374 (2004).
8. Bidet, M. *et al.* Clinical and molecular characterization of a cohort of 161 unrelated women with nonclassical congenital adrenal hyperplasia due to 21-hydroxylase deficiency and 330 family members. *J. Clin. Endocrinol. Metab.* **94,** 1570–1578 (2009).
9. Lajić, S. & Wedell, A. An intron 1 splice mutation and a nonsense mutation (W23X) in CYP21 causing severe congenital adrenal hyperplasia. *Hum. Genet.* **98,** 182–184 (1996).

10. Di Pasquale, L. *et al.* Novel nonsense mutation (W22X) in CYP21A2 gene causing salt-wasting congenital adrenal hyperplasia in a compound heterozygous girl. *J. Endocrinol. Invest.* **30,** 806–807 (2007).

11. Ezquieta, B., Oyarzábal, M., Jariego, C. M., Varela, J. M. & Chueca, M. A novel frameshift mutation in the first exon of the 21-OH gene found in homozygosity in an apparently nonconsanguineous family. *Horm. Res.* **51,** 135–141 (1999).

12. Lau, I. F. *et al.* H28+C insertion in the CYP21 gene: a novel frameshift mutation in a Brazilian patient with the classical form of 21-hydroxylase deficiency. *J. Clin. Endocrinol. Metab.* **86,** 5877–80 (2001).

13. Kharrat, M. *et al.* A novel 13-bp deletion in exon 1 of CYP21 gene causing severe congenital adrenal hyperplasia. *Diagn. Mol. Pathol.* **14,** 250–252 (2005).

14. Tardy, V. *et al.* Phenotype-genotype correlations of 13 rare CYP21A2 mutations detected in 46 patients affected with 21-hydroxylase deficiency and in one carrier. *J. Clin. Endocrinol. Metab.* **95,** 1288–1300 (2010).

15. Tardy, V. Gene symbol: CYP21A2. Disease: steroid 21-hydroxylase deficiency. *Hum. Genet.* **119,** 363 (2006).

16. Zeng, X. *et al.* Detection and assignment of CYP21 mutations using peptide mass signature genotyping. *Mol. Genet. Metab.* **82,** 38–47 (2004).

17. Brønstad, I. *et al.* Functional studies of novel CYP21A2 mutations detected in Norwegian patients with congenital adrenal hyperplasia. *Endocr. Connect.* **3,** 67–74 (2014).

18. Loidi, L. *et al.* High variability in CYP21A2 mutated alleles in Spanish 21-hydroxylase deficiency patients, six novel mutations and a founder effect. *Clin. Endocrinol. (Oxf).* **64,** 330–336 (2006).

19. Tardy, V. T. V & Morel, Y. Gene symbol: CYP21A2. *Hum. Genet.* **121,** 293 (2007).

20. Baradaran-Heravi, A. *et al.* Three novel CYP21A2 mutations and their protein modelling in patients with classical 21-hydroxylase deficiency from northeastern Iran. *Clin. Endocrinol. (Oxf).* **67,** 335–341 (2007).

21. Krone, N., Braun, A., Roscher, A. A. & Schwarz, H. P. A novel frameshift mutation (141delT) in exon 1 of the 21-hydroxylase gene (CYP21) in a patient with the salt wasting form of congenital adrenal hyperplasia. Mutation in brief no. 255. Online. *Hum. Mutat.* **14,** 90–91 (1999).

22. Concolino, P., Minucci, A., Mello, E., Zuppi, C. & Capoluongo, E. A new CYP21A2 nonsense mutation causing severe 21-hydroxylase deficiency. *Clin. Chem. Lab. Med.* **47,** 824–825 (2009).

23. Soardi, F. C. *et al.* Inhibition of CYP21A2 enzyme activity caused by novel missense mutations identified in Brazilian and Scandinavian patients. *J. Clin. Endocrinol. Metab.* **93,** 2416–2420 (2008).

24. Tardy, V. T. V. Gene symbol: CYP21A2. *Hum. Genet.* **121,** 292–293 (2007).

25. Ohlsson, G., Müller, J., Skakkebaek, N. E. & Schwartz, M. Steroid 21-hydroxylase deficiency: mutational spectrum in Denmark, three novel mutations, and in vitro expression analysis. *Hum. Mutat.* **13,** 482–486 (1999).

26. Wang, R. *et al.* 21-Hydroxylase deficiency-induced congenital adrenal hyperplasia in 230 Chinese patients: Genotype–phenotype correlation and identification of nine novel mutations. *Steroids* **108,** 47–55 (2016).
27. Nunez, B. S., Lobato, M. N., White, P. C. & Meseguer, a. Functional analysis of four CYP21 mutations from spanish patients with congenital adrenal hyperplasia. *Biochem. Biophys. Res. Commun.* **262,** 635–637 (1999).
28. Krone, N., Riepe, F. G., Grötzinger, J., Partsch, C.-J. & Sippell, W. G. Functional characterization of two novel point mutations in the CYP21 gene causing simple virilizing forms of congenital adrenal hyperplasia due to 21-hydroxylase deficiency. *J. Clin. Endocrinol. Metab.* **90,** 445–454 (2005).
29. New, M. I. *et al.* Genotype-phenotype correlation in 1,507 families with congenital adrenal hyperplasia owing to 21-hydroxylase deficiency. *Proc. Natl. Acad. Sci. U. S. A.* **110,** 2611–2616 (2013).
30. Krone, N., Roscher, A. A., Schwarz, H. P. & Braun, A. Comprehensive analytical strategy for mutation screening in 21-hydroxylase deficiency. *Clin. Chem.* **44,** 2075–2082 (1998).
31. Nikoshkov, A., Lajic, S., Holst, M., Wedell, A. & Luthman, H. Synergistic effect of partially inactivating mutations in steroid 21-hydroxylase deficiency. *J. Clin. Endocrinol. Metab.* **82,** 194–199 (1997).
32. Higashi, Y., Tanae, A., Inoue, H. & Fujii-Kuriyama, Y. Evidence for frequent gene conversion in the steroid 21-hydroxylase P-450(C21) gene: implications for steroid 21-hydroxylase deficiency. *Am. J. Hum. Genet.* **42,** 17–25 (1988).
33. Haider, S. *et al.* Structure-phenotype correlations of human CYP21A2 mutations in congenital adrenal hyperplasia. *Proc. Natl. Acad. Sci. U. S. A.* **110,** 2605–2610 (2013).
34. Concolino, P. *et al.* Two novel CYP21A2 missense mutations in Italian patients with 21-hydroxylase deficiency: Identification and functional characterisation. *IUBMB Life* **61,** 229–235 (2009).
35. Riepe, F. G. *et al.* Functional and Structural Consequences of a Novel Point Mutation in the CYP21A2 Gene Causing Congenital Adrenal Hyperplasia: Potential Relevance of Helix C for P450 Oxidoreductase-21-Hydroxylase Interaction. *J. Clin. Endocrinol. Metab.* **93,** 2891–2895 (2008).
36. Massimi, A. *et al.* Functional and Structural Analysis of Four Novel Mutations of CYP21A2 Gene in Italian Patients with 21-Hydroxylase Deficiency. *Horm. Metab. Res.* **46,** 515–520 (2014).
37. Krone, N. *et al.* Genotype-phenotype correlation in 153 adult patients with congenital adrenal hyperplasia due to 21-hydroxylase deficiency: analysis of the United Kingdom Congenital adrenal Hyperplasia Adult Study Executive (CaHASE) cohort. *J. Clin. Endocrinol. Metab.* **98,** E346–354 (2013).
38. Milacic, I. *et al.* Molecular genetic study of congenital adrenal hyperplasia in Serbia: novel p.Leu129Pro and p.Ser165Pro CYP21A2 gene mutations. *J. Endocrinol. Invest.* **38,** 1199–1210 (2015).
39. Taboas, M. *et al.* Functional studies of p.R132C, p.R149C, p.M283V, p.E431K, and a novel c.652-2A>G mutations of the CYP21A2 gene. *PLoS One* **9,** e92181 (2014).

40. Barbaro, M. *et al.* Functional studies of CYP21A2 mutants complement structural and clinical predictions of disease severity in CAH. *Clin. Endocrinol. (Oxf).* **76,** 764–766 (2012).
41. Chu, X. *et al.* Functional consequences of a novel point mutation in the CYP21A2 gene identified in a Chinese Han patient with nonclassic 21-hydroxylase deficiency. *Clin. Endocrinol. (Oxf).* **80,** 927–928 (2013).
42. Janner, M., Pandey, A. V, Mullis, P. E. & Flück, C. E. Clinical and biochemical description of a novel CYP21A2 gene mutation 962_963insA using a new 3D model for the P450c21 protein. *Eur. J. Endocrinol.* **155,** 143–151 (2006).
43. Robins, T. *et al.* Characterization of novel missense mutations in CYP21 causing congenital adrenal hyperplasia. *J. Mol. Med. (Berl).* **85,** 247–255 (2006).
44. Vrzalová, Z. *et al.* Identification of CYP21A2 mutant alleles in Czech patients with 21-hydroxylase deficiency. *Int. J. Mol. Med.* **26,** 595–603 (2010).
45. Grischuk, Y. *et al.* Four novel missense mutations in the CYP21A2 gene detected in Russian patients suffering from the classical form of congenital adrenal hyperplasia: identification, functional characterization, and structural analysis. *J. Clin. Endocrinol. Metab.* **91,** 4976–4980 (2006).
46. Witchel, S. F., Smith, R. & Suda-Hartman, M. Identification of CYP21 mutations, one novel, by single strand conformational polymorphism (SSCP) analysis. Mutations in brief no. 218. Online. *Hum. Mutat.* **13,** 172 (1999).
47. Billerbeck, A. E. C. *et al.* Three novel mutations in CYP21 gene in Brazilian patients with the classical form of 21-hydroxylase deficiency due to a founder effect. *J. Clin. Endocrinol. Metab.* **87,** 4314–4317 (2002).
48. Barbaro, M. *et al.* Functional studies of two novel and two rare mutations in the 21-hydroxylase gene. *J. Mol. Med. (Berl).* **84,** 521–528 (2006).
49. Higashi, Y., Hiromasa, T., Tanae, A., Miki, T. & Nakura, J. Effects of Individual Mutations in the P-450 (C21) Pseudogene on the P-450 (C21) Activity and Their Distribution in the Patient Genomes of. **644,** 638–644 (1991).
50. Stikkelbroeck, N. M. M. L. *et al.* CYP21 gene mutation analysis in 198 patients with 21-hydroxylase deficiency in The Netherlands: six novel mutations and a specific cluster of four mutations. *J. Clin. Endocrinol. Metab.* **88,** 3852–3859 (2003).
51. Concolino, P. *et al.* p.H282N and p.Y191H: 2 novel CYP21A2 mutations in Italian congenital adrenal hyperplasia patients. *Metabolism.* **61,** 519–524 (2012).
52. Nikoshkov, A. *et al.* Naturally Occurring Mutants of Human Steroid 21-Hydroxylase (P450c21) Pinpoint Residues Important for Enzyme Activity and Stability. *J. Biol. Chem.* **273,** 6163–6165 (1998).
53. Speiser, P. W., New, M. I. & White, P. C. Molecular Genetic Analysis of Nonclassic Steroid 21-Hydroxylase Deficiency Associated with HLA-B14,DR1. *N. Engl. J. Med.* **319,** 19–23 (1988).

54. Girgis, R., Ajamian, F. & Metcalfe, P. A previously undescribed mutation detected by sequence analysis of CYP21A2 gene in an infant with salt wasting congenital adrenal hyperplasia. *J. Pediatr. Endocrinol. Metab.* **26,** 205 (2013).
55. Concolino, P. *et al.* Functional analysis of two rare CYP21A2 mutations detected in Italian patients with a mildest form of congenital adrenal hyperplasia. *Clin. Endocrinol. (Oxf).* **71,** 470–476 (2009).
56. Krone, N. *et al.* Three novel point mutations of the CYP21 gene detected in classical forms of congenital adrenal hyperplasia due to 21-hydroxylase deficiency. *Exp. Clin. Endocrinol. Diabetes* **114,** 111–117 (2006).
57. Ezquieta, B. *et al.* Gene conversion (655G splicing mutation) and the founder effect (Gln318Stop) contribute to the most frequent severe point mutations in congenital adrenal hyperplasia (21-hydroxylase deficiency) in the Spanish population. *Clin. Genet.* **62,** 181–188 (2002).
58. Barbaro, M. *et al.* In vitro functional studies of rare CYP21A2 mutations and establishment of an activity gradient for nonclassic mutations improve phenotype predictions in congenital adrenal hyperplasia. *Clin. Endocrinol. (Oxf).* **82,** 37–44 (2014).
59. Robins, T., Barbaro, M., Lajic, S. & Wedell, A. Not All Amino Acid Substitutions of the Common Cluster E6 Mutation in CYP21 Cause Congenital Adrenal Hyperplasia. **90,** 2148–2153 (2005).
60. Kirac, D. *et al.* The Frequency and the Effects of 21-Hydroxylase Gene Defects in Congenital Adrenal Hyperplasia Patients. *Ann. Hum. Genet.* **78,** 399–409 (2014).
61. Koyama, S., Toyoura, T., Saisho, S., Shimozawa, K. & Yata, J. Genetic analysis of Japanese patients with 21-hydroxylase deficiency: identification of a patient with a new mutation of a homozygous deletion of adenine at codon 246 and patients without demonstrable mutations within the structural gene for CYP21. *J. Clin. Endocrinol. Metab.* **87,** 2668–2673 (2002).
62. Concolino, P., Mello, E., Zuppi, C. & Capoluongo, E. Molecular diagnosis of congenital adrenal hyperplasia due to 21-hydroxylase deficiency: an update of new CYP21A2 mutations. *Clin. Chem. Lab. Med.* **48,** 1057–1062 (2010).
63. Loke, K. Y., Lee, Y. S., Lee, W. W. & Poh, L. K. Molecular analysis of CYP-21 mutations for congenital adrenal hyperplasia in Singapore. *Horm. Res.* **55,** 179–184 (2001).
64. Finkielstain, G. P. *et al.* Comprehensive genetic analysis of 182 unrelated families with congenital adrenal hyperplasia due to 21-hydroxylase deficiency. *J. Clin. Endocrinol. Metab.* **96,** E161–172 (2011).
65. Bleicken, C. *et al.* Functional characterization of three CYP21A2 sequence variants (p.A265V, p.W302S, p.D322G) employing a yeast co-expression system. *Hum. Mutat.* **30,** E443–450 (2008).
66. Wu, DA; Chung, B. Mutations of P45Oc21 (Steroid 21-Hydroxylase) at Cys428, Val281, and Serf" Result in Complete, Partial, or No Loss of Enzymatic Activity, Respectively. *J. Clin. Invest.* **88,** 519–523 (1991).
67. Lajić, S., Robins, T., Krone, N., Schwarz, H. P. & Wedell, a. CYP21 mutations in simple virilizing congenital adrenal hyperplasia. *J. Mol. Med. (Berl).* **79,** 581–586 (2001).

68. Ezquieta, B. *et al.* Non-classical 21-hydroxylase deficiency in children: association of adrenocorticotropic hormone-stimulated 17-hydroxyprogesterone with the risk of compound heterozygosity with severe mutations. *Acta Paediatr.* **91,** 892–898 (2002).
69. Levo, A. & Partanen, J. Novel nonsense mutation (W302X) in the steroid 21-hydroxylase gene of a Finnish patient with the salt-wasting form of congenital adrenal hyperplasia. *Hum. Mutat.* **9,** 363–365 (1997).
70. Lajić, S. *et al.* Novel Mutations in CYP21 Detected in Individuals with Hyperandrogenism. *J. Clin. Endocrinol. Metab.* **87,** 2824–2829 (2002).
71. Dolzan, V. *et al.* Mutational spectrum of steroid 21-hydroxylase and the genotype-phenotype association in Middle European patients with congenital adrenal hyperplasia. *Eur. J. Endocrinol.* **153,** 99–106 (2005).
72. Lee, H. H. *et al.* Identification of four novel mutations in the CYP21 gene in congenital adrenal hyperplasia in the Chinese. *Hum. Genet.* **103,** 304–310 (1998).
73. Deneux, C. *et al.* Phenotype-genotype correlation in 56 women with nonclassical congenital adrenal hyperplasia due to 21-hydroxylase deficiency. *J. Clin. Endocrinol. Metab.* **86,** 207–13 (2001).
74. Bojunga, J. *et al.* Structural and functional analysis of a novel mutation of CYP21B in a heterozygote carrier of 21-hydroxylase deficiency. *Hum. Genet.* **117,** 558–564 (2005).
75. Globerman, H., Amor, M., Parker, K. L., New, M. I. & White, P. C. Nonsense mutation causing steroid 21-hydroxylase deficiency. *J. Clin. Invest.* **82,** 139–144 (1988).
76. Bernal González, C., Fernández Salas, C., Martínez, S. & Ezquieta Zubicaray, B. [Premature androgenetic alopecia in adult male with nonclassic 21-OH deficiency. A novel nonsense CYP21A2 mutation (Y336X) in 2 affected siblings]. *Med. Clin. (Barc).* **127,** 617–621 (2006).
77. Helmberg, A., Tusie-Luna, M. T., Tabarelli, M., Kofler, R. & White, P. C. R339H and P453S: CYP21 mutations associated with nonclassic steroid 21-hydroxylase deficiency that are not apparent gene conversions. *Mol. Endocrinol.* **6,** 1318–1322 (1992).
78. Krone, N. *et al.* The residue E351 is essential for the activity of human 21-hydroxylase: evidence from a naturally occurring novel point mutation compared with artificial mutants generated by single amino acid substitutions. *J. Mol. Med. (Berl).* **83,** 561–568 (2005).
79. Carvalho, D. F. *et al.* Molecular CYP21A2 Diagnosis in 480 Brazilian Patients with Congenital Adrenal Hyperplasia Before Newborn Screening Introduction. *Eur. J. Endocrinol.* 1–21 (2016).
80. Abid, F. *et al.* CYP21A2 gene mutation analysis in Moroccan patients with classic form of 21-hydroxylase deficiency: high regional prevalence of p.Q318X mutation and identification of a novel p.L353R mutation. *Clin. Chem. Lab. Med.* **46,** 1707–1713 (2008).
81. Krone, N., Braun, a, Roscher, a a, Knorr, D. & Schwarz, H. P. Predicting phenotype in steroid 21-hydroxylase deficiency? Comprehensive genotyping in 155 unrelated, well defined patients from southern Germany. *J. Clin. Endocrinol. Metab.* **85,** 1059–1065 (2000).

82. Lajić, S. *et al.* A cluster of missense mutations at Arg356 of human steroid 21-hydroxylase may impair redox partner interaction. *Hum. Genet.* **99,** 704–709 (1997).

83. Chiou, SH; Hu, M. and B. C. C. A missense mutation at Ile172----Asn or A Missense Mutation at Ile172 + Asn or Arg35s + Trp Causes Steroid. *J. Biol. Chem.* **265,** 3549–3552. (1990).

84. Levo, A. & Partanen, J. Novel mutations in the human CYP21 gene. *Prenat. Diagn.* **21,** 885–9 (2001).

85. Hsu, N.-C., Guzov, V. M., Hsu, L.-C. & Chung, B. Characterization of the consequence of a novel Glu-380 to Asp mutation by expression of functional P450c21 in Escherichia coli. *Biochim. Biophys. Acta - Protein Struct. Mol. Enzymol.* **1430,** 95–102 (1999).

86. Wasniewska, M. *et al.* Novel mutation of CYP21A2 gene (N387K) affecting a non-conserved amino acid residue in exon 9. *J Endocrinol Invest* **32,** 633 (2009).

87. Wedell, A. & Luthman, H. Steroid 21-hydroxylase deficiency: two additional mutations in salt-wasting disease and rapid screening of disease-causing mutations. *Hum. Mol. Genet.* **2,** 499–504 (1993).

88. Yu, Y. *et al.* Molecular characterization of 25 Chinese pedigrees with 21-hydroxylase deficiency. *Genet. Test. Mol. Biomarkers* **15,** 137–142 (2011).

89. Carvalho, B., Marques, C. J., Carvalho, D., Barros, A. & Carvalho, F. Novel human pathological mutations. Gene symbol: CYP21A2. Disease: adrenal hyperplasia. *Hum. Genet.* **127,** 482–483 (2010).

90. Jiang, L. *et al.* Identification and functional characterization of a novel mutation P459H and a rare mutation R483W in the CYP21A2 gene in two Chinese patients with simple virilizing form of congenital adrenal hyperplasia. *J. Endocrinol. Invest.* **35,** 485–489 (2012).

91. Minutolo, C. *et al.* Structure-based analysis of five novel disease-causing mutations in 21-hydroxylase-deficient patients. *PLoS One* **6,** e15899 (2011).

92. Ordoñez-Sánchez, M. L. *et al.* Molecular genetic analysis of patients carrying steroid 21-hydroxylase deficiency in the Mexican population: identification of possible new mutations and high prevalence of apparent germ-line mutations. *Hum. Genet.* **102,** 170–177 (1998).

93. Di Pasquale, G. *et al.* Salt wasting phenotype in a compound heterozygous girl with P482S mutation associated with anovel mutation of CYP21 gene (Q481P). *J. Endocrinol. Invest.* **28,** 1038–1039 (2005).

94. Wedell, A., Ritzén, E. M., Haglund-Stengler, B. & Luthman, H. Steroid 21-hydroxylase deficiency: three additional mutated alleles and establishment of phenotype-genotype relationships of common mutations. *Proc. Natl. Acad. Sci. U. S. A.* **89,** 7232–7236 (1992).

95. Rodrigues, N. R. *et al.* Molecular characterization of the HLA-linked steroid 21-hydroxylase B gene from an individual with congenital adrenal hyperplasia i. **6,** 1653–1661 (1987).

96. Arendse, L., Blundell, T. L. & Blackburn, J. Combining in silico protein stability calculations with structure-function relationships to explore the effect of polymorphic variation on cytochrome P450 drug metabolism. *Curr. Drug Metab.* **14,** 745–763 (2013).

Table S2: Calculated ΔΔG and predicted activity of CYP21A2 SNPs putatively involved in protein destabilization.

Variant	In silico activity (%)	Reference	Variant	In silico activity (%)	Reference
p.P34L	67.2	rs200648381:C>T	**p.W253R**	100	rs759857632:T>C
p.D43N	100	rs762507423:G>A	**p.R254K**	100	rs559386220:G>A
p.D43G	100	rs764569922:A>G	**p.D255E**	0.01	rs775688953:C>A
p.R75K	100	rs368330593:G>A	**p.M256T**	20.2	rs762929624:T>C
p.E78D	11.3	rs762330375:G>T	**p.M260V**	75.9	rs767558605:A>G
p.D87N	100	rs767973196:G>A	**p.P267L**	100	rs142028935:C>T
p.D87G	100	rs750793252:A>G	**p.S268G**	100	rs752306014:A>G
p.P105T	11.9	rs531645802:C>A	**p.S273Y**	100	rs78884659:C>A
p.D106N	100	rs774531624:G>A	**p.L276S**	1.5	rs747482395:C>A
p.T123I	100	rs566065375:C>T	**p.A284P**	0.02	rs775570889:G>C
p.I131M	100	rs746395253:C>G	**p.A285T**	21.1	rs570785206:G>A
p.D133Y	100	rs776029298:G>T	**p.V305A**	100	rs568758408:T>C
p.E136V	78.3	rs768973843:A>T	**p.H310N**	100	rs770059546:C>A
p.R149S	100	rs577450124:C>A	**p.P311S**	66.2	rs536088585:C>T
p.R149H	100	rs760710835:A>G	**p.I313L**	43.9	rs376415981:A>C
p.M150V	93.9	rs769769128:A>G	**p.D322H**	100	rs142058202:G>C
p.G155S	65.2	rs541292262:G>A	**p.E324K**	100	rs150804717:G>A
p.E163A	7.9	rs767249456:A>C	**p.E324G**	100	rs745826667:A>G
p.F164V	1.8	rs755674550:T>G	**p.G326S**	100	rs769730855:G>A
p.S170L	100	rs754305318:G>T	**p.A329V**	100	rs774151904:C>T
p.L175H	1.6	rs779166970:T>A	**p.S331G**	100	rs767161968:A>G
p.T176N	100	rs748501160:C>A	**p.R333W**	100	rs749917058:C>T
p.D184N	100	rs745933819:G>A	**p.R333Q**	100	rs756621561:G>A
p.Y190N	100	rs768524764:T>A	**p.Y336H**	100	rs754209599:T>C
p.T200A	100	rs760425681:A>G	**p.T348S**	30.5	rs370908729:C>G
p.S202G	88.4	rs372964292:A>G	**p.I349M**	100	rs144104274:C>T
p.H203Y	100	rs764468228:T>C	**p.A350T**	65.2	rs768586475:G>A
p.I212T	21.1	rs764468228:T>C	**p.A350V**	18.4	rs774242376:C>T
p.P219L	75.9	rs770752895:C>T	**p.D377N**	100	rs528524868:G>A
p.N220S	100	rs780780640:A>G	**p.H392Q**	38.9	rs745358717:C>G
p.G222C	14.7	rs769293380:G>T	**p.T396M**	100	rs568795145:C>T
p.L223P	100	rs775023782:T>C	**p.V397I**	100	rs763395640:G>A
p.D234E	100	rs10947229:T>G	**p.E399G**	100	rs764542690:A>G
p.D234N	100	rs774835577:G>A	**p.H402R**	100	rs774540237:A>G
p.R242S	78.3	rs150496227:G>C	**p.L419P**	100	rs761006767:T>C
p.H244R	0.05	rs553299378:A>G	**p.V441L**	100	rs750190235:G>C
p.S247R	100	rs772680196:C>G	**p.T443I**	13.4	rs755816115:C>T
p.G251S	17.9	rs182942340:G>A	**p.P465S**	20.2	rs748620874:C>T

In silico enzymatic activities of SNPs lacking functional assays and presumed to affect protein stability were analyzed. Predicted activities were calculated from the fitting of the bovine based model (Figure 1) using the estimated **ΔΔG** of each of the variants (Table S1) and are expressed relative to the wild type protein considered as 100%. SNPs were retrieved from http://www.ncbi.nlm.nih.gov/projects/SNP/

Table S3: Double mutants' stability calculations

Group	Mutation 1	ΔΔG	Mutation 2	ΔΔG	Sum of individual ΔΔG	Double mutants' Foldx calculation
A	p.G375S	1.32	p.M473I	0.28	1.6	1.6
	p.C147R	1.47	p.D407N	0.14	1.61	1.62
	p.I230T	0.6	p.V304M	1.01	1.61	2.17
	p.G178A	1.35	p.M473I	0.28	1.63	1.65
	p.C147R	1.47	p.R149C	0.2	1.67	2.31
	p.A391T	1.56	p.D407N	0.14	1.7	1.71
	p.C147R	1.47	p.M473I	0.28	1.75	1.76
	p.R149C	0.2	p.A391T	1.56	1.76	1.73
	p.I194N	1.22	p.I230T	0.6	1.82	2.03
	p.A391T	1.56	p.M473I	0.28	1.84	1.79
	p.I230T	0.6	p.G375S	1.32	1.92	1.93
	p.G178A	1.35	p.I230T	0.6	1.95	1.93
	p.C147R	1.47	p.I230T	0.6	2.07	2.49
	p.I230T	0.6	p.A391T	1.56	2.16	2.45
	p.V304M	1.01	p.G375S	1.32	2.33	2.39
	p.G178A	1.35	p.V304M	1.01	2.36	2.37
	p.C147R	1.47	p.V304M	1.01	2.48	2.57
	p.I194N	1.22	p.G375S	1.32	2.54	2.39
	p.G178A	1.35	p.I194N	1.22	2.57	2.58
	p.V304M	1.01	p.A391T	1.56	2.57	2.97
	p.G178A	1.35	p.G375S	1.32	2.67	2.59
	p.C147R	1.47	p.I194N	1.22	2.69	2.75
	p.I194N	1.22	p.A391T	1.56	2.78	2.73
	p.C147R	1.47	p.G375S	1.32	2.79	2.7
	p.C147R	1.47	p.G178A	1.35	2.82	2.83
	p.G375S	1.32	p.A391T	1.56	2.88	2.8
	p.G178A	1.35	p.A391T	1.56	2.91	2.93
	p.C147R	1.47	p.A391T	1.56	3.03	3.04
B	p.P105L	-0.57	p.M283V	1.8	1.23	0.54
	p.P105L	-0.57	p.Y191H	1.86	1.29	1.28
	p.P105L	-0.57	p.R149P	1.99	1.42	1.53
C	p.I194N	1.22	p.V304M	1.01	2.23	1.5
D	p.P105L	-0.57	p.P453S	2.18	1.61	1.55
	p.M283V	1.8	p.D407N	0.14	1.94	1.5
	p.R149C	0.2	p.M283V	1.8	2	1.09
	p.M283V	1.8	p.M473I	0.28	2.08	1.5
	p.M283V	1.8	p.V304M	1.01	2.81	1.07
	p.Y191H	1.86	p.V304M	1.01	2.87	1.58
	p.R149P	1.99	p.V304M	1.01	3	1.33

Double mutants *in cis* were grouped in different categories: **Group A**: none of the mutations

exceeds the cut off value but their sum indeed does. The combined analysis by FoldX gives similar results. **Group B**: only one of the mutations exceeds the cut off value, but combined with another mutation, the sum drops below the cut off. The combined analysis by FoldX gives similar results. **Group C**: a negative synergistic effect in which none of the mutations exceeds the cut off value, the sum does, but their combined analysis by FoldX results in a lower value. **Group D:** a negative synergistic effect in which one of the mutations exceeds the cut off value, their sum also does, but its combined analysis by FoldX gives a lower value. ΔΔG is expressed in kcal/mol relative to free energy of the wild type protein.

Table S4: Stability calculations of mutant-SNP *in cis*

Group	Mutation	ΔΔG	SNP	ΔΔG	Sum of individual ΔΔG	Double variants' FoldX calculation
A	p.C147R	1.47	p.A159T	0.39	1.86	2.19
	p.I194N	1.22	p.K102R	0.81	2.03	2.24
	p.V304M	1.01	p.K102R	0.81	1.82	1.65
	p.G375S	1.32	p.A159T	0.39	1.71	1.61
	p.G375S	1.32	p.A265V	0.47	1.79	1.7
	p.G375S	1.32	p.V249A	0.45	1.77	1.68
	p.G375S	1.32	p.S268T	0.74	2.06	1.98
	p.G375S	1.32	p.S268M	0.4	1.72	1.65
	p.I194N	1.22	p.A159T	0.39	1.61	1.67
	p.C147R	1.47	p.K102R	0.81	2.28	2.22
	p.C147R	1.47	p.S268M	0.4	1.87	1.81
	p.A391T	1.56	p.K102R	0.81	2.37	2.32
	p.G375S	1.32	p.K102R	0.81	2.13	2.09
	p.A391T	1.56	p.A159T	0.39	1.95	1.99
	p.A391T	1.56	p.S268M	0.4	1.96	1.93
	p.I194N	1.22	p.S268T	0.74	1.96	1.93
	p.G178A	1.35	p.S268M	0.4	1.75	1.77
	p.G178A	1.35	p.V249A	0.45	1.8	1.78
	p.G178A	1.35	p.K102R	0.81	2.16	2.15
	p.G178A	1.35	p.S268T	0.74	2.09	2.1
	p.A391T	1.56	p.S268C	0.3	1.86	1.87
	p.I194N	1.22	p.V249A	0.45	1.67	1.68
	p.A391T	1.56	p.A265V	0.47	2.03	2.04
	p.A391T	1.56	p.D183E	0.12	1.68	1.69
	p.G178A	1.35	p.S268C	0.3	1.65	1.66
	p.C147R	1.47	p.S268T	0.74	2.21	2.2
	p.A391T	1.56	p.V249A	0.45	2.01	2.02
	p.G178A	1.35	p.A159T	0.39	1.74	1.74
	p.C147R	1.47	p.A265V	0.47	1.94	1.94
	p.G178A	1.35	p.A265V	0.47	1.82	1.82
	p.I194N	1.22	p.A265V	0.47	1.69	1.69
	p.C147R	1.47	p.S268C	0.3	1.77	1.77
	p.V304M	1.01	p.S268T	0.74	1.75	1.75
	p.A391T	1.56	p.S268T	0.74	2.3	2.3
	p.C147R	1.47	p.V249A	0.45	1.92	1.92
**B**	p.M283V	1.8	p.V211M	-0.91	0.89	-0.15
	p.P453S	2.18	p.V211M	-0.91	1.27	1.37
	p.Y191H	1.86	p.V211M	-0.91	0.95	1.01
	p.R149P	1.99	p.V211M	-0.91	1.08	1.06
**C**	p.G375S	1.32	p.S268C	0.3	1.62	1.51
	p.I194N	1.22	p.S268M	0.4	1.62	1.55

Group	Mutation	ΔΔG	SNP	ΔΔG	Sum of individual ΔΔG	Double variants' FoldX calculation
D	p.M283V	1.8	p.V249A	0.45	2.25	1.49
	p.M283V	1.8	p.A159T	0.39	2.19	1.44
	p.M283V	1.8	p.S268C	0.3	2.1	1.37
	p.M283V	1.8	p.A265S	-0.03	1.77	1.08
	p.M283V	1.8	p.S268M	0.4	2.2	1.54
	p.M283V	1.8	p.D183E	0.12	1.92	1.41
E	p.P30L	2.43	p.V211M	-0.91	1.52	2.11
F	p.I230T	0.6	p.K102R	0.81	1.41	2.25
	p.C147R	1.47	p.D183E	0.12	1.59	1.99

The combination of a mutation and a SNP *in cis* were grouped in different categories: **Group A:** neither the mutation nor the SNP exceeds the cut off value, but their sum indeed does. The combined analysis by FoldX gives similar results. **Group B**: the mutation exceeds the cut off value but combined with the SNP their sum drops below the cut-off value. The combined analysis by FoldX gives similar results. **Group C**: a negative synergistic effect in which neither the mutation nor the SNP exceeds the cut off value, the sum does, but their combined analysis by FoldX results in a lower value. **Group D**: a negative synergistic effect in which the mutation exceeds the cut off value, when combined with a SNP the sum also does, but their combined analysis by FoldX gives a lower value. **Group E**: a positive synergistic effect in which the sum of the mutation and the SNP does not exceed the cut-off value, but their combined analysis with FoldX indeed does. **Group F**: neither the mutation and the SNP, nor their sum, exceeds the cut-off value, but FoldX nevertheless predicts a synergistic. **ΔΔG** is expressed in kcal/mol relative to free energy of the wild type protein.

Table S5: Genotypes and phenotypes of 21-hydroxylase patients bearing novel mutations

Patient	Novel mutation	Accession number	Genotype[a]	Gender	Phenotype
1	g.910T>A	KU533637	c.290-13A/C>G/ **p.L107Q**	M	NC-SV[c]
2	g.956T>G	KU533638	c.290-13A/C>G/ **p.L122R**	F	NC[d]
3	g.986G>A	KU533639	p.V281L/p.[**R132H** ;V281L]	F	NC
4	g.2262C>T	KU533640	p.[V281L;**P335L**]/ Conv or del	F	NC
5	g.2834dupC	KU987427	c.290-13A/C>G-p.Q318*/**p.H466fs**[b]	M	SW

a: Maternal/Paternal allele. Only mothers from patients 1 and 4 were available. For patients 2, 3 and 5, segregation of the alleles was assumed. Novel mutations are displayed in bold. **b**: This patient has one chromosome with 2 copies of the *CYP21A2* gene. Long range PCRs positioned the novel p.H466fs mutation and the p.Q318* in the centromeric copies. The c.290-13A/C>G-p.Q318* haplotype has been previously described as an haplotype found in chromosomes bearing a duplicated *CYP21A2* gene, each copy with one of the mutations [1,2] . **c:** The distinction between SV and NC form is quite clear in most cases for females as the appearance of virilized external genitalia characterizes the SV form. In males, this distinction could be more difficult. **d:** This patient has hormone levels compatible with a SV form of the disease (17-OHP=260 ng/mL; Testosterone: 6.7 ng/mL) and external genitalia with clitoromegaly size of 3 cm. Nevertheless, the labia majora and minora are normally developed with no fusion abnormality. She was classified as a NC CAH based on the absence of ambiguous genitalia. M: male; F: Female. NC: Nonclassical; SV: Simple virilizing; SW: Salt wasting; Conv or del: large gene conversion or deletion.

REFERENCES

1. Wedell A., Stengler B. & Luthman H. Characterization of mutations on the rare duplicated C4/CYP21 haplotype in steroid 21-hydroxylase deficiency. *Hum Genet* **94**:50–54 (1994).
2. Loidi L., *et al*. High variability in CYP21A2 mutated alleles in Spanish 21-hydroxylase deficiency patients, six novel mutations and a founder effect. *Clin Endocrinol (Oxf)* **64**:330–336 (2006).

FIGURE S1: **Partial alignment analyses of CYP21A2 proteins from different mammalian species.**

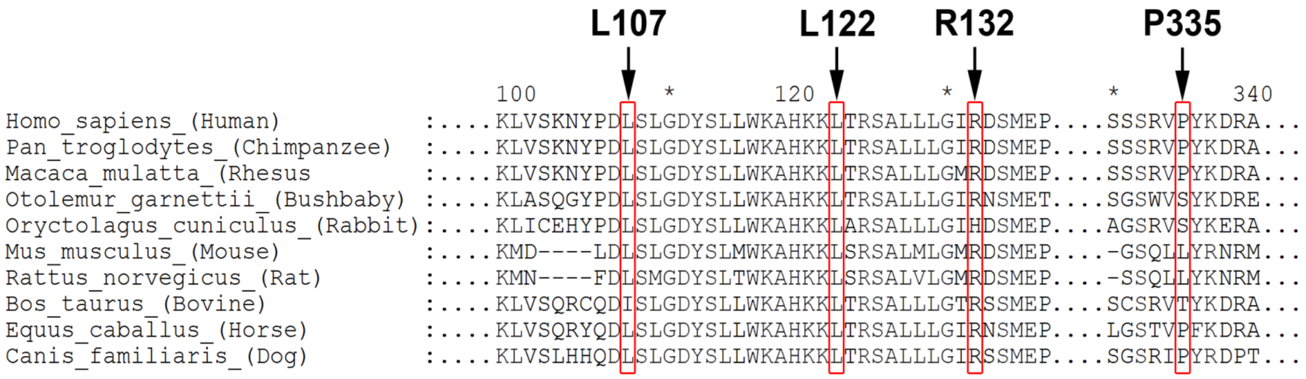

Similarities between CYP21 proteins were assessed using sequences from different mammalian species. The location of each point mutation found in patients from our cohort is labelled and indicated by a red rectangle.

FIGURE S2: **Structural analysis of novel point substitution using the bovine CYP21A2 structure based-model.**

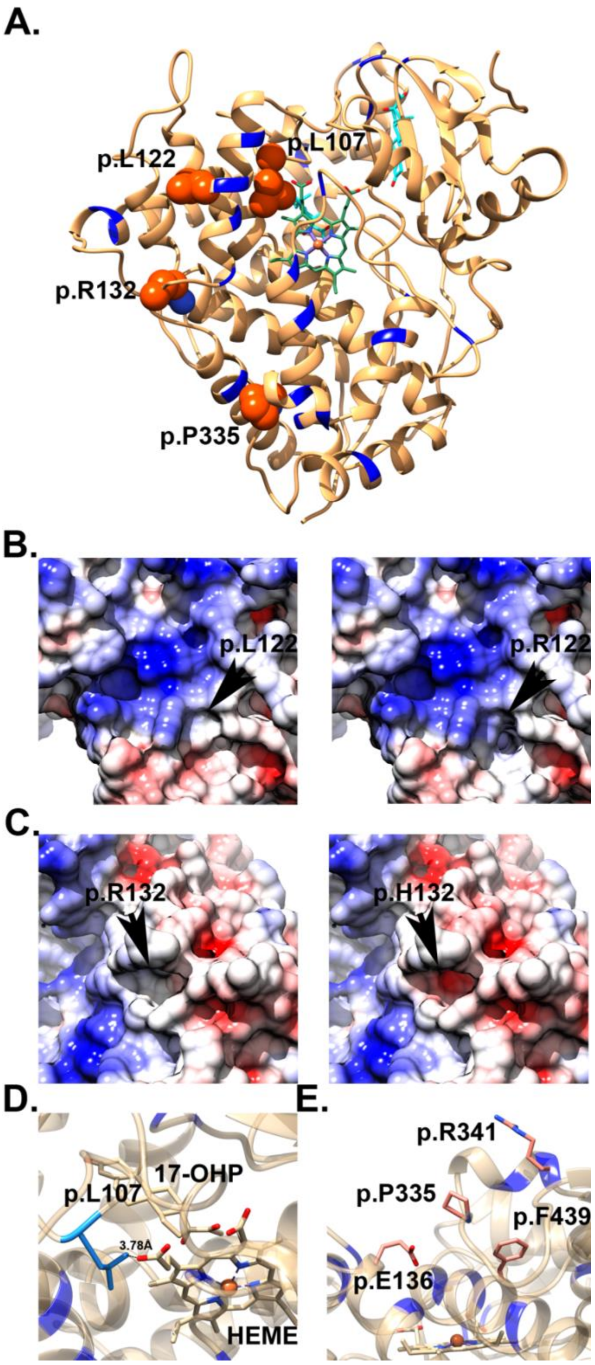

A: Cartoon representation of the residues involved in the novel point mutations found. Residues are labeled and highlighted by orange spheres. Heme cofactor is depicted in sticks. Residues involved in POR interaction are depicted in blue. **B and C:** Differences in electrostatic surface upon mutations. Surface electrostatics of the wild type L122 and R122 as well as R122 and H132 mutants are represented. Residues are indicated by arrows. Acidic regions are depicted in

red and basic ones in blue. **D:** Cartoon representation of the residue L107 in the structure. Residue L107 (in light blue sticks) points towards the heme´s propionate moiety at a distance of 3.87Å. HEME: heme group; 17-OHP: 17-hydroxyprogesterone. **E:** Cartoon representation of the residue P335 in the structure. Residues located nearby in the 3D structure are also shown.
